# Plasma proteomics stratification identifies phospholamban R14del carriers at risk for disease progression

**DOI:** 10.1093/cvr/cvag089

**Published:** 2026-04-25

**Authors:** Frederik E Deiman, Taner Arslan, Remco de Brouwer, Ian Henry, Lars Löfgren, Anders Cavallin, Filippos Michopoulos, Ralf Nilsson, Marcus Henricsson, Christine Ahlstrom, Pia Davidsson, Nils Bomer, Niels Grote Beverborg, Peter van der Meer

**Affiliations:** Department of Cardiology, University of Groningen, University Medical Center Groningen, 9713 GZ Groningen, The Netherlands; AstraZeneca, Research and Early Development, Cardiovascular, Renal and Metabolism (CVRM), BioPharmaceuticals R&D, SE-431 83 Gothenburg, Sweden; Department of Cardiology, University of Groningen, University Medical Center Groningen, 9713 GZ Groningen, The Netherlands; AstraZeneca, Research and Early Development, Cardiovascular, Renal and Metabolism (CVRM), BioPharmaceuticals R&D, SE-431 83 Gothenburg, Sweden; AstraZeneca, Research and Early Development, Cardiovascular, Renal and Metabolism (CVRM), BioPharmaceuticals R&D, SE-431 83 Gothenburg, Sweden; AstraZeneca, Research and Early Development, Cardiovascular, Renal and Metabolism (CVRM), BioPharmaceuticals R&D, SE-431 83 Gothenburg, Sweden; AstraZeneca, Research and Development, Oncology, Biologics Engineering and Oncology Targeted Discovery, BioPharmaceuticals R&D, Cambridge CB2 0AA, UK; AstraZeneca, Research and Early Development, Cardiovascular, Renal and Metabolism (CVRM), BioPharmaceuticals R&D, SE-431 83 Gothenburg, Sweden; AstraZeneca, Research and Early Development, Cardiovascular, Renal and Metabolism (CVRM), BioPharmaceuticals R&D, SE-431 83 Gothenburg, Sweden; AstraZeneca, Research and Early Development, Cardiovascular, Renal and Metabolism (CVRM), BioPharmaceuticals R&D, SE-431 83 Gothenburg, Sweden; AstraZeneca, Research and Early Development, Cardiovascular, Renal and Metabolism (CVRM), BioPharmaceuticals R&D, SE-431 83 Gothenburg, Sweden; Department of Cardiology, University of Groningen, University Medical Center Groningen, 9713 GZ Groningen, The Netherlands; Department of Cardiology, University of Groningen, University Medical Center Groningen, 9713 GZ Groningen, The Netherlands; Department of Cardiology, University of Groningen, University Medical Center Groningen, 9713 GZ Groningen, The Netherlands

**Keywords:** Heart failure, Cardiomyopathy, Phospholamban, Phospholamban R14del, Proteomics, Metabolomics, Lipidomics

## Abstract

**Aims:**

Incomplete penetrance is common in genetic cardiomyopathy, but poorly understood. Here, we investigate the circulating molecular signature in a cohort of patients with one specific phospholamban (PLN) p.Arg14del (R14del, R14^Δ/+^) pathogenic variant underlying R14^Δ/+^ cardiomyopathy and its association with disease variability and progression.

**Methods and results:**

Targeted proteomics, metabolomics, and lipidomics were performed on plasma from 87 R14^Δ/+^ carriers across the disease spectrum. Unsupervised clustering of plasma proteomics classified R14^Δ/+^ carriers into clusters, which were evaluated using clinical data, including heart failure (HF) symptoms, echocardiographic parameters, and clinical follow-up. Metabolomics and lipidomics data were integrated. Five clusters of R14^Δ/+^ carriers were identified based on plasma proteomics (*N* = 2612 proteins). Clusters 3, 4, and 5 were enriched for higher N-terminal pro-B-type natriuretic peptide levels, and lower left ventricular ejection fraction, compared with Clusters 1 and 2. Ninety-six out of 148 metabolites were differentially expressed across the clusters. Levels of symmetric dimethylarginine, N-acetyl aspartate, cis-aconitic acid, S-adenosyl-L-methionine, acadesine, and succinate were elevated in disease condition Clusters 3, 4, and 5. Levels of energy metabolism–related metabolites (i.e. adenosine triphosphate and nicotinamide) were elevated in Clusters 1, 3, and 4 and correlated strongly with apoptosis markers, indicating ongoing cardiac damage. Clusters 1 and 2 represent seemingly asymptomatic R14^Δ/+^ carriers with Cluster 1 suspected at risk for cardiac damage due to elevated apoptosis markers. Cluster 3 shows an intermediate phenotype, and Clusters 4 and 5 consist of R14^Δ/+^ carriers with end-stage HF. Clinical follow-up confirmed Cluster 1 at risk for R14^Δ/+^ cardiomyopathy progression due to increased adverse events (HF hospitalization, all-cause mortality, or cardiac device implantation).

**Conclusion:**

Molecular profiling of R14^Δ/+^ carriers reveals subgroups with very distinct risk profiles. Early markers of cardiac damage suggest that stratification may enable timely identification of high-risk individuals and improve understanding of disease variability.

## Graphical Abstract

**Graphical Abstract:**
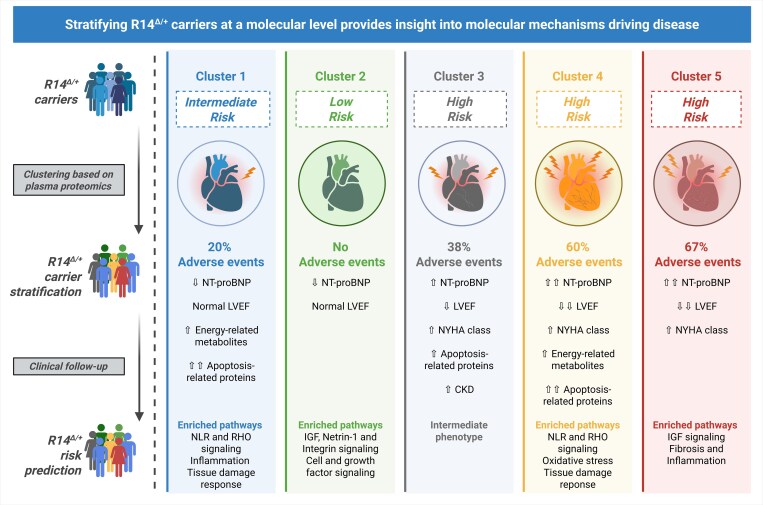

**Time for primary review: 29 days**

## 1. Introduction

Phospholamban (PLN) p.Arg14del (R14del, R14^Δ/+^) is a pathogenic variant that can lead to heart failure (HF). The variant is known to play an important role in the development of dilated and arrhythmogenic cardiomyopathy.^1, 2^ The R14^Δ/+^ phenotype is characterized by a high degree of cardiac fibrosis, reduced electrocardiogram potentials, fibrofatty replacement, and PLN protein aggregation.^1, 2^ There are no effective treatment options available for R14^Δ/+^. Currently, patients with R14^Δ/+^ cardiomyopathy are treated with standard HF medication that suppress the symptoms of HF, but do not address the underlying pathophysiology of the disease. As a result, patients show a high rate of disease progression and often require a left ventricular assist device (LVAD) or a heart transplant.^3^

To date, the biological pathways contributing to the onset and progression of R14^Δ/+^ cardiomyopathy remain poorly understood. Recent studies have identified promising precision medicine modalities.^4–7^ However, the successful translation of these therapies into clinical practice requires a deeper understanding of the molecular mechanisms driving disease development. In the context of precision medicine, the greatest benefit of treatment can be achieved when applied at the onset of disease. R14^Δ/+^ cardiomyopathy exhibits incomplete penetrance, with disease manifestation occurring at varying ages, ranging from early adulthood to late life.^1,2^ This variability suggests the involvement of additional molecular factors that influence disease progression.

The heart exerts a significant influence on circulating biomolecules,^8^ including proteins, metabolites, and lipids. Investigating changes in these molecular pathways across the R14^Δ/+^disease spectrum may provide insight into the mechanisms underlying cardiomyopathy progression. Stratifying patients based on molecular profiles rather than only clinical characteristics may enable targeted treatment strategies. For R14^Δ/+^ cardiomyopathy, understanding the molecular heterogeneity that underlies variable disease expression is critical for the design of future therapeutic trials. By defining biologically distinct patient subgroups, molecular stratification can help identify those at highest risk for progression and guide the application of emerging therapies, such as gene therapy.^9^ This approach ultimately bridges molecular pathophysiology with clinical translation, moving towards individualized treatment for genetic heart disease such as R14^Δ/+^.

There is a critical need to understand the biological pathways that drive the onset and progression of R14^Δ/+^ cardiomyopathy. In this work, we investigate the molecular pathways associated with R14^Δ/+^ cardiomyopathy using plasma proteomics, highlighting dysregulated processes that may contribute to disease progression. We show that stratifying a specific HF population of R14^Δ/+^ carriers, before the appearance of clinical signs and symptoms at a molecular level, offers a promising tool for early and targeted therapeutic interventions for improved patient outcomes.

## 2. Methods

### 2.1 Plasma sample collection

For this study, samples collected within the DECIPHER-PLN cohort were used (ClinicalTrials.gov Id: NCT04978987).^31^ Plasma samples from 88 R14^Δ/+^ carriers across the R14^Δ/+^ disease spectrum were included. All participants provided written informed consent prior to inclusion in the study. Briefly, Plasma collection of R14^Δ/+^ carriers was ethically approved by the scientific advisory board of the University Medical Center Groningen and was conducted in accordance with the principles of the declaration of Helsinki (Protocol number 2020.326 and 2020.327, UMCG Research Register no. 202000351, ABR-number NL73976.042.20). Plasma was collected by drawing blood into BD Vacutainer EDTA Tubes (BD Medical). Within 30 min after blood drawing, the tube was spun down at 2000xg for 10 min at room temperature. The upper plasma phase was collected and stored at −80°C, until used for proteomics, metabolomics, and lipidomics. Plasma sample collection was performed before LVAD implantation. In addition, none of the patients had a cardiac resynchronization therapy device.

### 2.2 Targeted proteomics

Proximity Extension Assay (PEA) technology (Olink Explore 3072) was performed for the ethylenediaminetetraacetic acid (EDTA) plasma samples, quantifying 2926 proteins. The analysis was carried out according to the manufacturer's instructions by Olink Proteomics (Uppsala, Sweden). Targeted proteomics is based on PEA,^32^ coupled with readout via next-generation sequencing. Final data consisted of 2611 proteins. The raw output data were quality controlled, normalized, and converted into normalized protein expression, the proprietary unit of relative abundance in Olink. Data normalization was performed using an internal extension control and an external plate control to adjust for intra- and inter-run variation. During data processing, background correction, log_2_ transformation, and normalization were performed using Olink software. Proteins with expression levels below the limit of detection (LOD) in more than 50% of samples were removed, and expression values below LOD converted to LOD/sqrt(2), as recommended by Olink.

### 2.3 Targeted metabolomics

In total, 148 metabolites were quantified as the observed detector response (arbitrary units) in EDTA plasma using 2 separate targeted metabolomics methods. Identities of metabolites were based on comparison of retention times and mass transitions of analytes in the samples and in reference solution containing all measured analytes, in total over 300 known metabolites (see Supplementary material online, *Table S6*). Targeted metabolomics in positive electrospray ionization tandem mass spectrometry mode (TMET+) was used to quantify positively charged metabolites after protein precipitation of plasma samples and chromatographic separation based on hydrophilic interaction liquid chromatography. Sixty two metabolites were successfully quantified in plasma samples using TMET+. Method details are given in Supplementary material online (Targeted Metabolomics TMET+ Method). Targeted metabolomics in negative electrospray ionization time-of-flight mass spectrometry (TMET−) was used to quantify negatively charged metabolites after protein precipitation and chromatographic separation based on ion-pair chromatography. 86 metabolites were successfully quantified in plasma samples using TMET−. Methods for TMET+ and TMET− were also reported in a previous study on plasma metabolites.^33^

### 2.4 Targeted lipidomics

Plasma lipids were extracted using the butanol:methanol method and then analysed using ultra-performance liquid chromatography coupled to tandem mass spectrometry.^34^ Deuterated internal standards were added during extraction. For sphingolipids, a Waters bridged ethylene hybrid (BEH) C8 column (2.1 × 100 mm, 1.7µm) was used with mobile phases consisting of water and acetonitrile as mobile phase A and acetonitrile:water as mobile phase B.^35^ In total approximately 40 molecular species were quantified from six different sphingolipid classes [dihydroceramides, ceramides, glucosylceramides, lactosylceramides, globotriaosylceramide (Gb3), and monosialodihexosyl-ganglioside]. The phospholipids were analysed with hydrophilic interaction liquid chromatography using a Waters BEH amide column (2.1 × 100 mm, 1.7 µm) and 95% acetonitrile with 5 mM ammonium formate as mobile Phase A and water with 10 mM ammonium formate as mobile Phase B.^36^ In total ∼40 different molecular species were quantified from 3 different lipid classes (phosphatidylcholines, phosphatidylethanolamines, and sphingomyelins). Quantification was made with a combination of internal and external standards.

### 2.5 Computational environment

All analyses were performed using R version 4.1. Standard and specialized R packages were used for data preprocessing, statistical analysis, and visualization.

### 2.6 Statistical analysis

All statistical analyses were conducted in R version 4.1. For group comparisons involving multiple clusters in the box plots, the Kruskal–Wallis test was used. Spearman correlation was applied to assess relationships between continuous variables. Hypergeometric test was performed to investigate any clinical variables enrichment across clusters. Adjusted *P*-values were computed using the Benjamini–Hochberg correction.

### 2.7 Unsupervised clustering analysis

Unbiased clustering analysis was performed on plasma protein data using consensus clustering.^37^ Hierarchical clustering was applied as the clustering method, with 1000 iterations to ensure stability. Spearman correlation was used as the distance metric. Clustering stability and the optimal number of clusters were determined based on consensus matrices and cumulative distribution function plots. Based on an explanatory analysis, one outlier sample was identified and removed from the dataset before any downstream analysis.

### 2.8 Dimensionality reduction and visualization

To visualize the distribution of samples in a two-dimensional space, PCA was performed. Samples were coloured according to their cluster assignments to assess the separation between identified clusters.

### 2.9 Differential expression analysis

To identify differentially expressed plasma proteins between clusters, the limma (Linear Models for Microarray Data) method was applied using a one vs. rest approach.^38^ This allowed for the comparison of each cluster against all others to determine upregulated and downregulated proteins. Proteins with an adjusted *P*-value <0.05 (Benjamini–Hochberg correction) were considered significant. Results were visualized using volcano plots to highlight significantly altered proteins and UpSet plots to show shared and unique differentially expressed proteins across clusters.

### 2.10 Pathway enrichment analysis

To identify enriched biological pathways, Reactome pathway analysis was performed using the clusterProfiler R package.^39^ Only upregulated proteins (adjusted *P*-value <0.05) from each cluster were used as input to focus on activated pathways, as including both up- and downregulated proteins can make it harder to interpret the direction of changes. The background proteins are the plasma proteins (*n* = 2611) from the Olink assay. To increase data transparency, pathway enrichment analysis of downregulated proteins is reported in Supplementary material online, *Figure S4*. The ‘tree’ function from the enrichplot package was applied to visualize relationships between enriched pathways, grouping similar pathways based on their functional similarity.

### 2.11 Medication burden analysis

Medication burden analysis was conducted using two complementary approaches: (i) Delta Akaike information criterion (AIC) analysis^40,41^ to identify proteins for clustering exclusion based on statistical significance of medication effects and (ii) Partial *R*^2^ analysis^42^ to quantify effect sizes and validate the selection criteria. Clustering stability was assessed using the ARI,^43^ which is a metric that measures the similarity between two clusters (or partitions) of the same data, correcting for chance agreement. A score of +1 indicates perfect agreement, while a score near zero suggests random similarity, and negative values approaching −1 indicate less similarity than expected by chance.

### 2.12 Integration of metabolomics and lipidomics data

To assess whether metabolites and lipid species were differentially abundant across clusters, a linear model was applied. For metabolites, estimated glomerular filtration rate values were included as a covariate to account for potential confounding effects. *P*-values from the linear model were used to assess statistical significance. For visualization, the median abundance of each analyte was calculated within each cluster. The minimum and maximum median values across clusters were then subtracted to quantify the range of variation. A scatter plot was generated, with the y-axis representing the *P*-value from the linear model and the x-axis representing the calculated difference (max−min of median values across clusters), highlighting analytes with significant and large abundance changes.

### 2.13 Clinical follow-up

The cumulative incidence of adverse events (AEs; HF hospitalization, device implantation and heart transplant/LVAD incidence, and all-cause mortality) was evaluated across the DECIPHER-PLN cohort using clinical data records after a median follow-up of 916 days. Risk scores were evaluated using cox regression analysis and plotted utilizing Kaplan–Meier curve.

## 3. Results

### 3.1 Unsupervised clustering based on the plasma proteome identified five R14^Δ/+^ clusters with distinct clinical profiles

**Figure 1:**
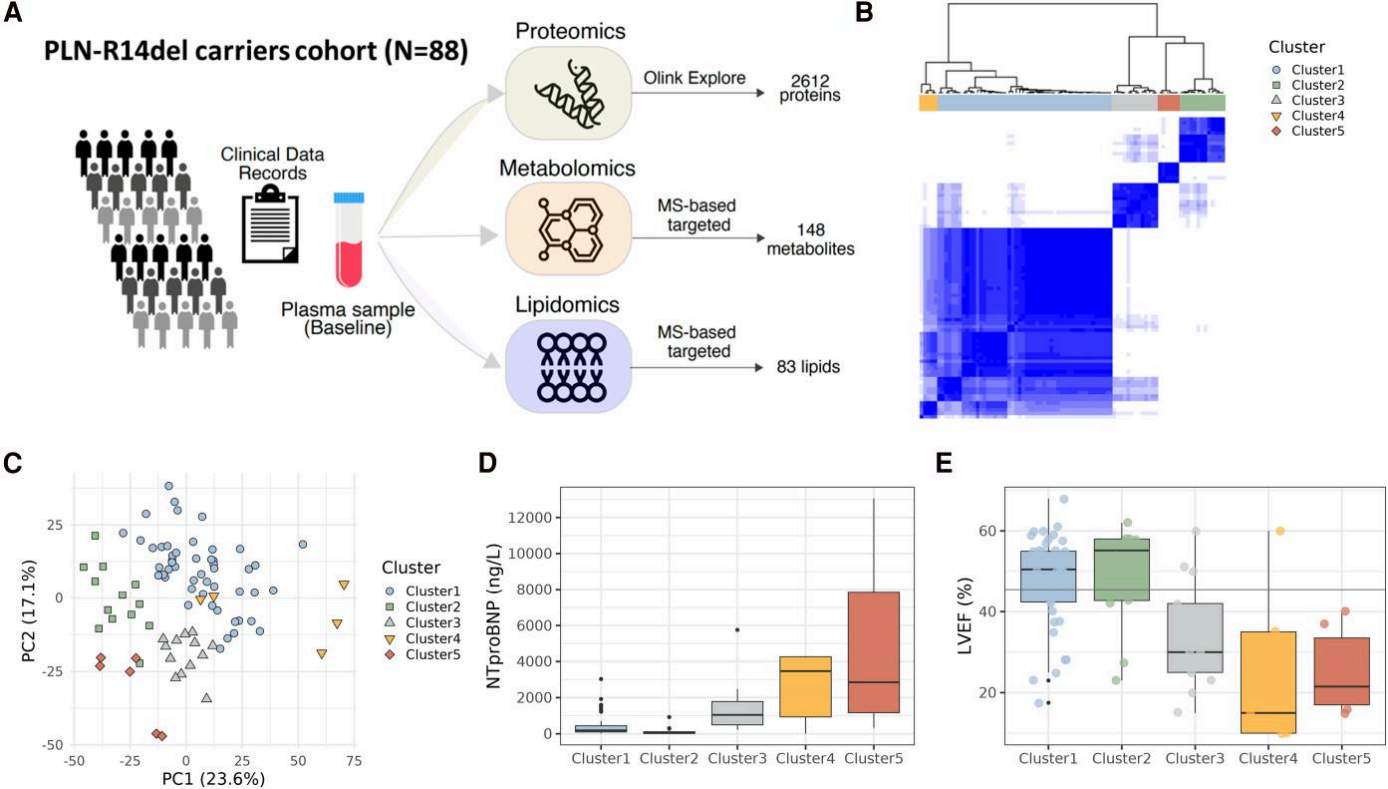
Unsupervised clustering identified five clusters with distinct plasma protein profiles. *A*) Workflow of the study; clinical data records and plasma samples are collected across the R14^Δ/+^ disease spectrum. Plasma samples of R14^Δ/+^ carriers (*N* = 88, biological replicates; no technical replicates) are subjected to targeted proteomics, metabolomics, and lipidomics to identify the molecular pathways associated with R14^Δ/+^ cardiomyopathy across the disease spectrum. One sample was excluded as an outlier prior to downstream analyses (*N* = 87). *B*) Unsupervised clustering of plasma proteomics. *C*) PCA of clusters. *D*) NT-proBNP levels in ng/L (*P*-value = 2.725e−06). *E*) LVEF (*P*-value = 2.348e−03). Significance was determined using Kruskal–Wallis test.

Unsupervised clustering of patients based on the plasma proteome could potentially identify subtypes of disease driven by molecular-level variations. For this purpose, plasma proteomics, based on OLINK Explore (*N* = 2612 proteins), was used for clustering (*Figure 1A*). Eighty-eight R14^Δ/+^ carriers were initially included with one exclusion due to being an outlier (see Supplementary material online, *Figure S1*). Five clusters were identified using unsupervised clustering (*Figure 1B*). Stability analysis demonstrated robust clustering with a mean item consensus score of 0.842, where 93% of samples demonstrated good-to-excellent stability (>0.7), validating their biological significance despite modest sample sizes. The five cluster solution represents the optimal balance between biological interpretability and statistical robustness, as confirmed by our stability analysis (see Supplementary material online, *Figure S2*). Principal component analysis (PCA) confirmed the observations of the unsupervised clustering analysis (*Figure 1C*). Targeted metabolomics and targeted lipidomics were integrated across the R14^Δ/+^ disease spectrum, and the patient clusters were evaluated using clinical data records (*Table 1*). The clusters did not differ significantly with respect to sex or age (see Supplementary material online, *Table S1*). Clusters 3, 4, and 5 are enriched with R14^Δ/+^ carriers with advanced disease, characterized by higher N-terminal pro-B-type natriuretic peptide (NT-proBNP) levels, lower left ventricular ejection fraction (LVEF), higher New York Heart Association (NYHA) class, and more previous hospitalizations due to HF, whereas Clusters 1 and 2 represented the least affected R14^Δ/+^ carriers (see Supplementary material online, *Table S1*). Clusters 4 and 5 had the highest NT-proBNP levels (*P*-value <0.001) and the lowest LVEF (*P*-value <0.001), whereas Cluster 3 presented moderate NT-proBNP and LVEF levels (*Figure 1D* and *E*). All patients with chronic kidney disease (*N* = 4) were clustered in Cluster 3 (see Supplementary material online, *Table S2*). Diuretics and mineralocorticoid receptor antagonists were enriched in Clusters 3, 4, and 5. Angiotensin receptor/neprilysin inhibitors (ARNIs) were enriched in Clusters 3 and 5, whereas sodium–glucose transport protein-2 inhibitors and inotropes were enriched in Clusters 4 and 5 (see Supplementary material online, *Table S3*).

**Table 1:** Baseline characteristics of R14^Δ/+^ clusters

Factor	Overall
*N*	87
Age, mean (SD)	54.46 (13.92)
Sex (male/female)	43 (49%)
Female	51% (44/87)
Male	49% (43/87)
BMI, mean (SD)	26.38 (4.19)
Pulse, mean (SD)	69.05 (13.58)
Systolic BP, mean (SD)	120.80 (18.79)
Diastolic BP, mean (SD)	75.26 (9.71)
NYHA, (N)	87
I	6,9% (6/87)
II	20% (17/87)
III	9,2% (8/87)
IV	5,7% (5/87)
No HF	59% (51/87)
Smoking, (*N*)	20 (24%)
Hypertension, (*N*)	5 (6%)
Hypercholesterolaemia, (*N*)	4 (5%)
Diabetes mellitus (*N*)	2 (2%)
Laboratory	
*N*	87
NT-proBNP (ng/L), median (IQR)	280.00 (89.00, 1220.00)
cardiac troponin T (cTNT) (ng/L), median (IQR)	13.00 (8.00, 20.00)
Echo	
*N*	76
LVEF, mean (SD)	46 (30, 55)
LVEF (missing)	11

Baseline clinical characteristics of R14^Δ/+^ carriers (*N* = 87 biological replicates; no technical replicates) included in the study. *N* represents the number of individual patients. Values are presented as mean ± SD, median (IQR), or number (%), as indicated.

BMI, body mass index; IQR, interquartile range; BP, blood pressure.

### 3.2 Plasma proteome–based pathways driving patient clustering across the R14^Δ/+^ spectrum

**Figure 2:**
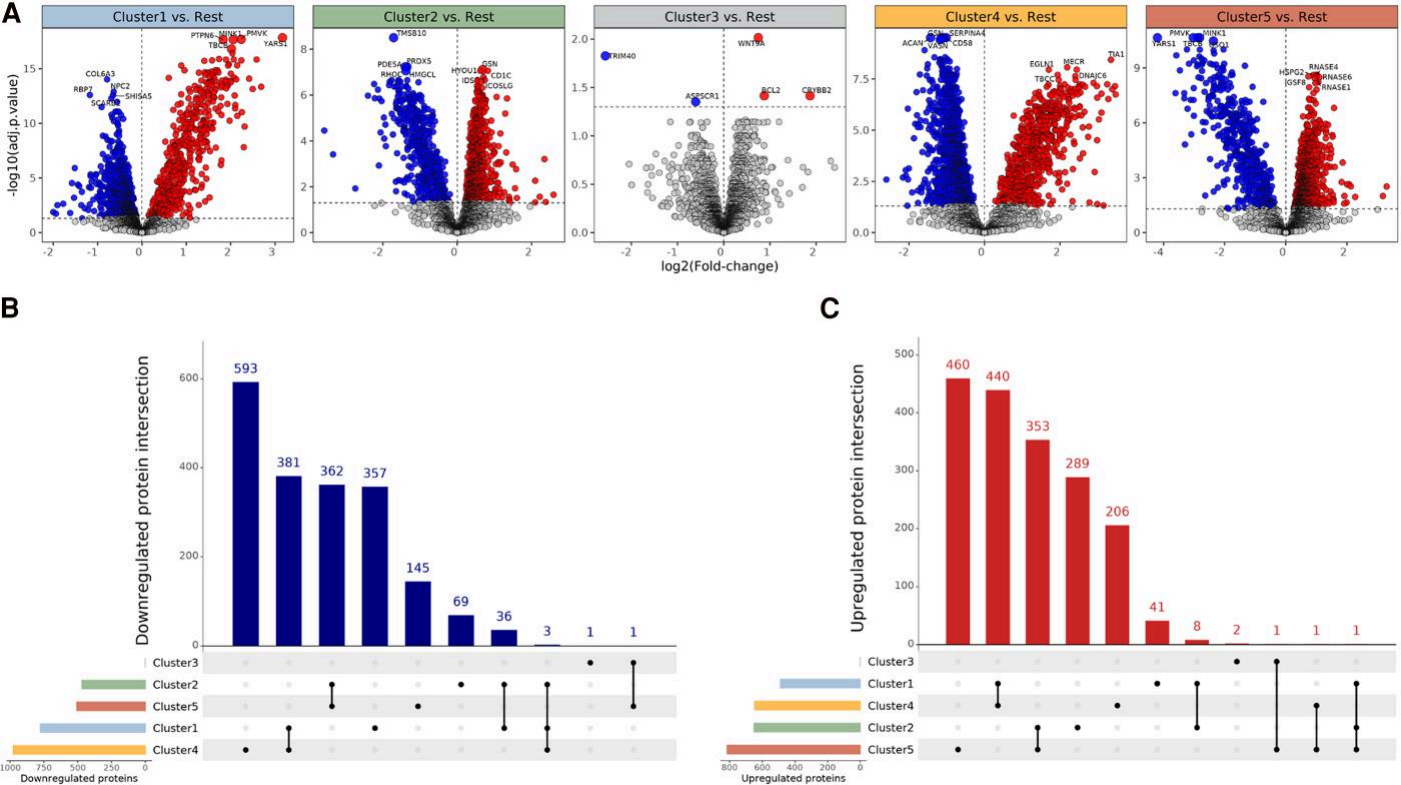
Differential analysis revealed distinct levels of plasma proteins across the clusters. *A*) Distinct proteins amongst each cluster using one vs. rest approach assessed with linear models (limma), with adjusted *P*-value <0.05, with Benjamini–Hochberg correction (N = 87 R14^Δ/+^ carriers). *B*) Downregulated protein intersection amongst R14^Δ/+^ clusters. *C*) Upregulated protein intersection amongst R14^Δ/+^ clusters.

Plasma proteins across the clusters were compared with one another and revealed distinct pathways associated with different clusters (*Figure 2A*), with distinct downregulated (*Figure 2B*) and upregulated proteins (*Figure 2C*). Cluster 1 showed 1267 proteins (777 downregulated and 490 upregulated). Pathway enrichment analysis of the 490 upregulated proteins revealed that Cluster 1 associated with Ras homologous guanosine triphosphatase (Rho GTPase) signalling, cell cycle and mitosis, immune and antiviral responses, and apoptosis and cell death pathways (see Supplementary material online, *Figure S3A*). Cluster 2 showed 1121 proteins (470 downregulated and 651 upregulated). Pathway enrichment analysis of the 651 upregulated proteins in Cluster 2 revealed enrichment of pathways related to extracellular matrix and cell interactions (integrin and netrin-1 signalling), haemostasis, clotting and growth factor (insulin-like growth factor 1) signalling (see Supplementary material online, *Figure S3B*). Cluster 3 showed five proteins (two downregulated and three upregulated) and was excluded from pathway enrichment analysis due to the low amount of differentially expressed plasma proteins. Cluster 4 showed 1624 proteins (977 downregulated and 647 upregulated). Pathway enrichment analysis of the 647 upregulated proteins in Cluster 4 revealed enrichment of pathways related to tissue damage, in particular oxidative stress, chemical stress response, cell cycle dysregulation, apoptosis, and signalling pathways that maintain cellular structure and function (see Supplementary material online, *Figure S3C*). Cluster 5 showed 1324 proteins (508 downregulated and 816 upregulated). Cluster 5 reflects pathways that are deeply involved in the pathological processes of HF, particularly those associated with tissue damage, fibrosis, immune response, and thrombosis (see Supplementary material online, *Figure S3D*). The overlap of up- and downregulated proteins between clusters reflects shared molecular pathways active across different phenotypic stages. For example, Clusters 1 (low NT-proBNP and preserved LVEF) and 4 (high NT-proBNP and reduced LVEF) showed substantial overlap in proteins related to apoptosis, oxidative stress, and tissue remodelling. These shared molecular pathways indicate that changes in Cluster 1 may involve activation of molecular programmes that are also prominent in advanced disease, such as Cluster 4.

### 3.3 Medication burden has minimal impact on proteomic disease clustering

**Figure 3:**
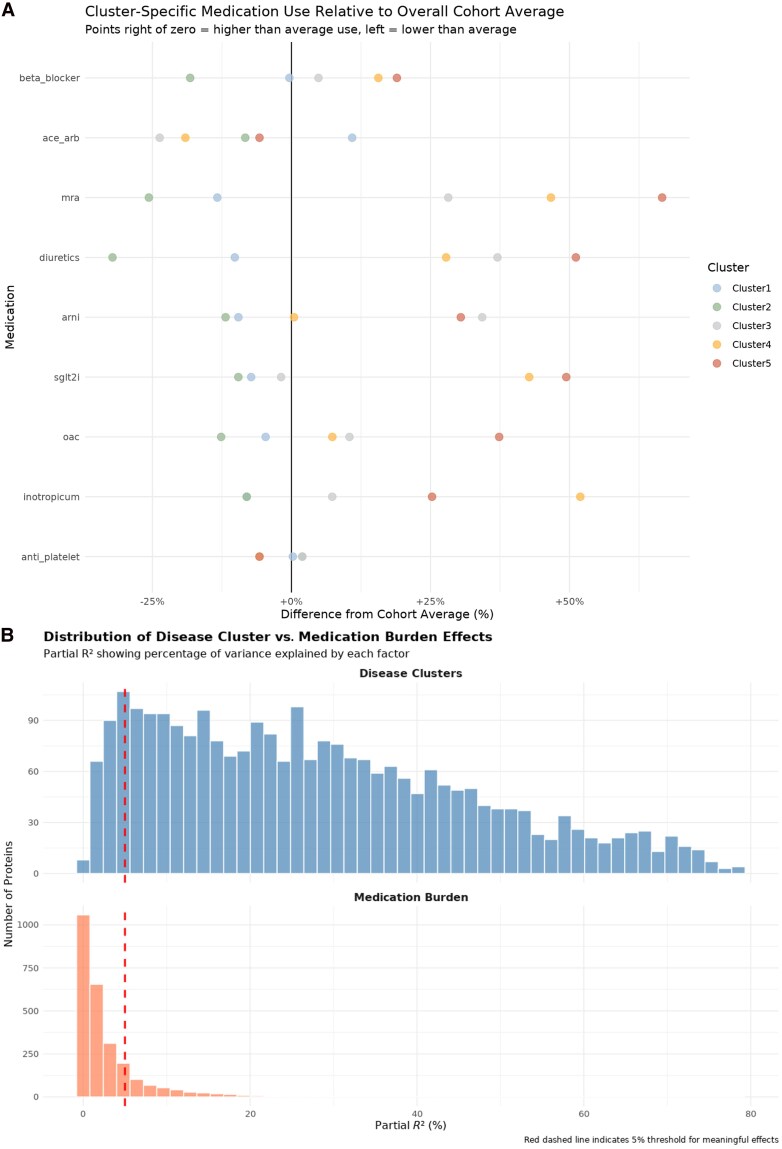
Distribution of disease cluster vs. medication burden effects on protein expression. *A*) Love plot showing cluster-specific medication use relative to the overall cohort average (*N* = 87 R14^Δ/+^ carriers). *B*) Dual histogram comparing the distribution of partial *R*^2^ values for disease cluster effects (blue, upper panel) vs. medication burden effects (coral, lower panel) across all 2611 proteins. Each histogram shows the percentage of protein expression variance explained by the respective factor. The red dashed vertical line indicates the 5% threshold for clinically meaningful effects. Disease cluster effects show a right-shifted distribution with higher variance explained (mean = 28.0%, median = 25.3%) compared with medication burden effects (mean = 2.9%, median = 1.3%). The dramatic difference in proteins exceeding the 5% threshold (2375 vs. 450 proteins) demonstrates that disease biology dominates the proteomic signature by approximately five-fold in terms of affected proteins and 10-fold in terms of effect magnitude. This visualization clearly illustrates that while medication effects are detectable, they represent a minority signal compared with the overwhelming influence of disease phenotypes on protein expression patterns.

Cluster-specific medication use relative to the overall cohort differed between clusters (*Figure 3A*). To assess the influence of concomitant medical therapies on the clustering, we conducted a comprehensive medication burden analysis. First, ΔAIC analysis identified 511 proteins (19.6% of total) whose expression was significantly influenced by medication burden. Second, partial *R*^2^ analysis showed that 450 of these 511 proteins (88%) had >5% variance explained by medication burden, while the remaining 61 proteins had smaller but statistically significant effects (mean 4.7% variance explained). Across all proteins, disease clusters explained substantially more variance than medication burden (mean partial *R*^2^: 28.0 vs. 2.9%), with 2375 proteins (91.0%) showing >5% disease cluster effects (*Figure 3B*; see Supplementary material online, *Table S4*). This comprehensive medication burden analysis demonstrates that medication burden represents one component of the proteomic variation, but it does not substantially alter the clustering results. Because the 511 medication-sensitive proteins showed evidence of medication influence, we evaluated them separately in a sensitivity analysis to determine whether their removal would affect clustering. Importantly, even within this subset, disease clusters still accounted for more variance than medication burden (30.6 vs. 9.8%; see Supplementary material online, *Table S5*). After excluding these 511 proteins, the resulting unsupervised clustering remained highly similar to the original [adjusted Rand index (ARI) = 0.837], indicating that medication-sensitive proteins do not meaningfully alter the patient stratification.

### 3.4 Metabolic derangements were apparent between clusters and correlated strongly with proteomic profiles of apoptosis

**Figure 4:**
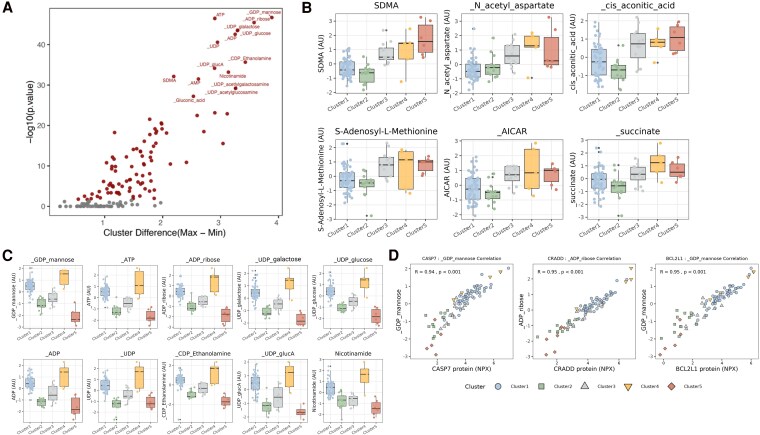
Targeted metabolomics identified metabolites associated to different R14^Δ/+^ disease clusters. *A*) Distinct metabolites based on cluster differences (*N* = 87 R14^Δ/+^ carriers), linear model with corrected creatinine implemented. Median metabolite levels were calculated across clusters and followed by finding maximum and minimum median levels. *B*) Distinct metabolite levels of SDMA, NAA, cis-aconitic acid, SAMe, AICAR, and succinate levels across R14^Δ/+^ clusters. *C*) Distinct nucleotide levels of GDP mannose, ATP, ADP-ribose, UDP-galactose, UDP-glucose, ADP, UDP, CDP-ethanolamine, UDP-glucuronic acid, and nicotinamide levels across the R14^Δ/+^ clusters. *D*) Correlation plot of nucleotide derivatives to apoptosis-related proteins CASP7, CRADD, and BCL2L1. Statistical significance was assessed using linear regression models.

Targeted metabolomics revealed that the levels of 96 out of 148 metabolites were significantly different across the clusters (*Figure 4A*). Specifically, the levels of symmetric dimethylarginine (SDMA), N-acetyl aspartate (NAA), cis-aconitic acid, S-adenosyl-L-methionine (SAMe), acadesine (AICAR), and succinate were found to be elevated in the disease Clusters 3, 4, and 5 (*Figure 4B*). Additionally, energy metabolism–related metabolites (nucleotide-derived compounds) were different between clusters. These nucleotide-derived compounds include guanosine diphosphate (GDP) mannose, adenosine triphosphate (ATP), adenosine diphosphate (ADP) ribose, uridine diphosphate (UDP) galactose, UDP-glucose, ADP, UDP, cytidine diphosphate (CDP), ethanolamine, UDP glucuronic acid (glucA), and nicotinamide. These nucleotides were found to be elevated in Cluster 4 (and to a lesser extent in Clusters 1 and 3), reflecting increased metabolic demand, and decreased in Cluster 5 (*Figure 4C*). These metabolites were found to correlate strongly with apoptosis-related proteins caspase 7 (CASP7), Caspase and RIP adaptor with death domain (CRADD), and protein phosphatase 1, regulatory subunit 52 (BCL2L1; *Figure 4D*).

### 3.5 Targeted lipidomics identified altered PC(16:0/22:2), PC(18:0/22:2), and 2-linoleoyl-sn-glycero-3-phosphoethanolamine levels in disease Cluster 4

**Figure 5:**
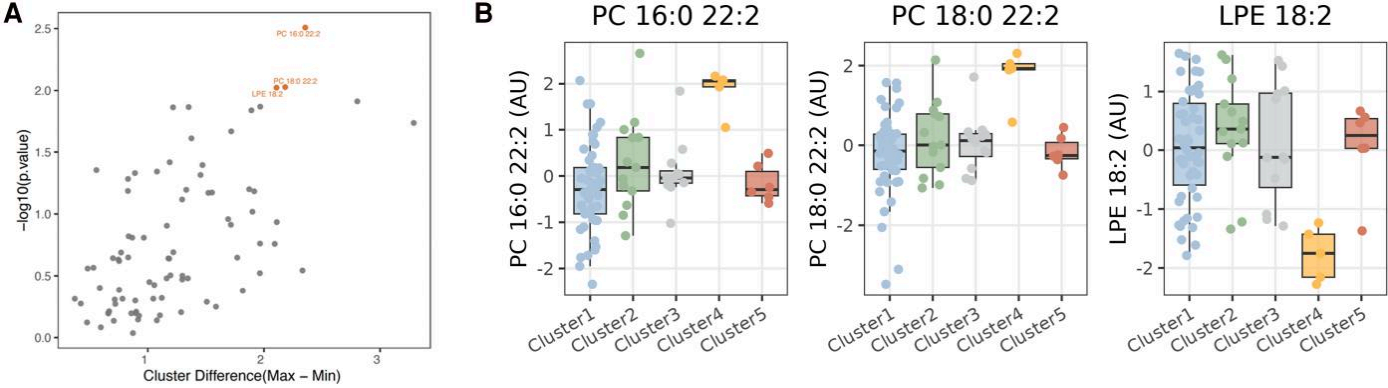
Targeted lipidomics identified lipids associated to different R14^Δ/+^ disease clusters. *A*) Distinct lipids based on cluster differences (*N* = 87 R14^Δ/+^ carriers). Median lipid levels were calculated across clusters and followed by finding maximum and minimum median levels. *B*) Distinct lipid levels of PC(16:0/22:2), PC(18:0/22:2), and LPE(18:2) across R14^Δ/+^ clusters. Statistical significance was assessed using linear regression models.

Of the 40 phospholipid species analysed, three showed significant differences between the clusters (*Figure 5A*). In disease Cluster 4, levels of phosphatidylcholine (PC) (16:0/22:2) (1-palmitoyl-2-docosadienoyl-sn-glycero-3-phosphocholine; C_46_H_88_NO_8_P) and PC(18:0/22:2) (1-stearoyl-2-docosadienoyl-sn-glycero-3-phosphocholine; C_48_H_92_NO_8_P) were increased, while 2-linoleoyl-sn-glycero-3-phosphoethanolamine [LPE(18:2); C_23_H_44_NO_7_P) levels were decreased (*Figure 5B*). Levels of the 40 glycosphingolipids were unchanged across the clusters.

### 3.6 Integration of clinical data records, proteomics, metabolomics, and lipidomics reveals healthy-like, disease progression, and end-stage clustering in R14^Δ/+^ patients

**Figure 6:**
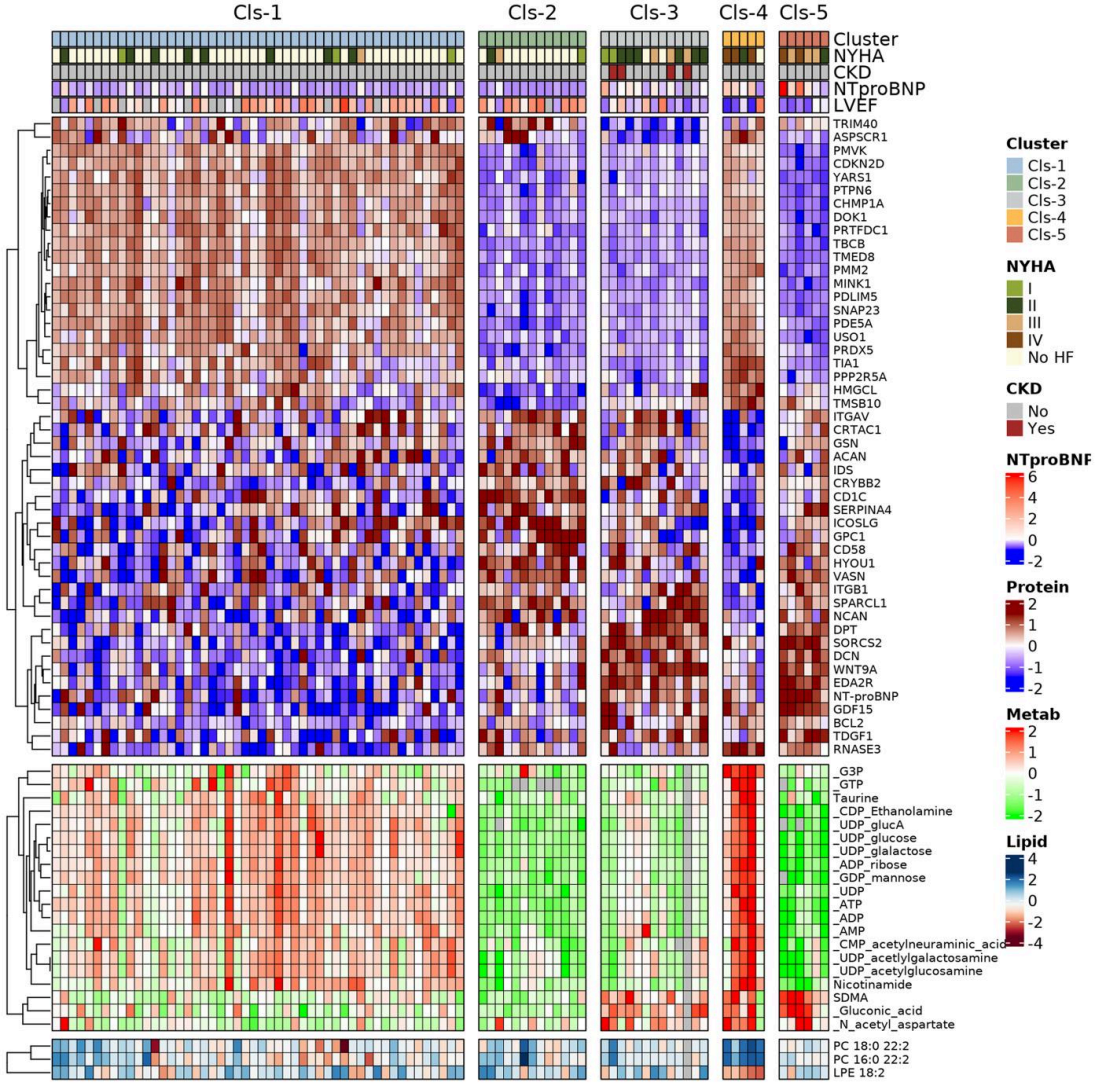
Comprehensive overview of distinct proteins, metabolites, lipids across the R14^Δ/+^ disease spectrum. Proteins, metabolites, and lipid species associated to different R14^Δ/+^ clusters (*N* = 87 R14^Δ/+^ carriers) identified utilizing unsupervised clustering.

By integrating multi-omics with clinical data records, clusters were associated to R14^Δ/+^ disease stage. Cluster 1 represents a cluster without HF and unaffected by R14^Δ/+^, hence lower NT-proBNP and higher LVEF. Interestingly, elevated energy metabolism–related metabolites that associated with apoptosis-related protein markers were observed. Cluster 2 represents a cluster predominantly free of HF (only 16% with HF symptoms), like Cluster 1 (18% HF symptoms) with lower NT-proBNP and higher LVEF, but vastly different plasma proteomic profile and unchanged energy metabolism–related metabolites. Cluster 3 represents a disease progession cluster enriched with NYHA II and end-stage R14^Δ/+^, with higher NT-proBNP and lower LVEF compared with Clusters 1 and 2, but lower than Clusters 4 and 5. Cluster 4 represents an end-stage R14^Δ/+^ cluster with NYHA IV, highest NT-proBNP, and lowest LVEF. Cluster 4 was enriched in pathways related to response to tissue damage (active cell growth, division, and repair processes pathways) and showed the highest plasma levels of ATP, ADP, and glucose metabolites. Cluster 5 represents an end-stage R14^Δ/+^ cluster with NYHA III and IV, highest NT-proBNP, and lowest LVEF, but vastly different plasma proteomic profile and unchanged energy metabolism–related metabolites when compared with Cluster 4. Cluster 5 was enriched in pathways related to tissue repair (fibrosis and inflammatory pathways). In total, several proteins (i.e. hypoxia upregulated 1, peroxiredoxin 5, charged multi-vesicular body protein 1a), metabolites (i.e. NAA, aconitic acid, and SAMe), and lipid species (PC16:0/22:2, PC18:0/22:2, and LPE 18:2) were identified as circulating biomolecules associating to the different R14^Δ/+^ clusters (*Figure 6*).

### 3.7 Evaluation of adverse events across R14^Δ/+^ clusters in clinical follow-up

**Figure 7:**
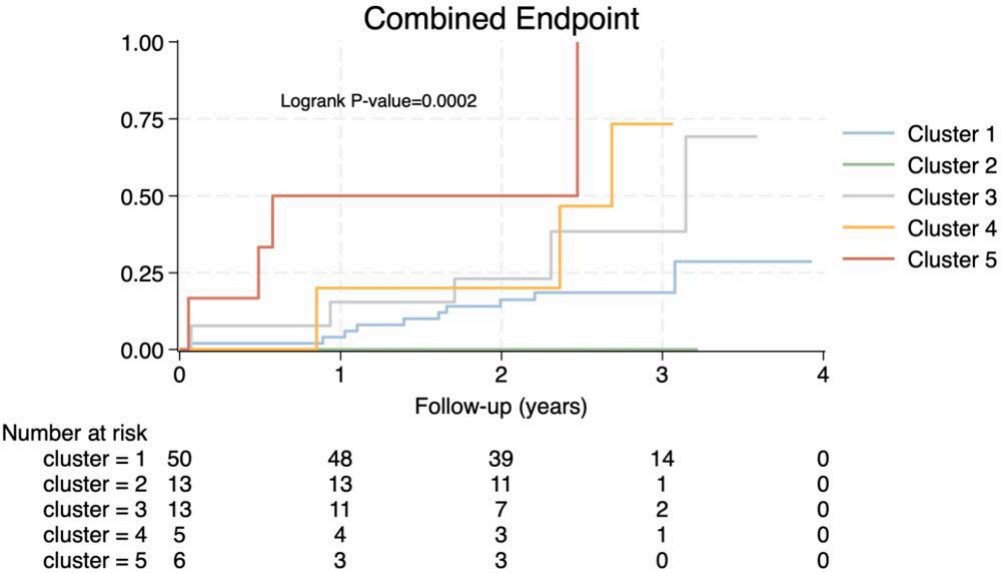
Evaluation of adverse events across the R14^Δ/+^ clusters. *A*) Kaplan–Meier plot and risk table revealing the cumulative incidence of adverse events (HF hospitalization, device implantation, heart transplantation, LVAD implantation, and all-cause mortality) across the R14^Δ/+^ clusters (*N* = 87 R14^Δ/+^ carriers) with a median follow-up of 914 days. Ten events were observed in Cluster 1 (first event after 23 days and last event after 1125 days). Zero events in Cluster 2. Five events in Cluster 3 (26–1149 days after inclusion). Three events in Cluster 4 (23–982 days after inclusion) and four events in Cluster 5 (20–903 days after inclusion). Statistical significance was determined using the log-rank test (*P* = 0.0002) across the clusters. *B*) Kaplan–Meier plot and risk table after combining the clusters with disease progression (increased NT-proBNP and NYHA Class and decreased LVEF) 3, 4, and 5 together (*P* = 0.0007 using log-rank test).

The cumulative incidence of adverse events (AEs; HF hospitalization, device implantation and heart transplant/LVAD incidence, and all-cause mortality) were evaluated across the R14^Δ/+^ clusters using clinical data records after a median follow-up of 916 days using Kaplan–Meier analysis. Clinical follow-up demonstrated that Cluster 2 (0% AEs) has a very low risk for adverse events, Cluster 1 (20% AEs) has intermediate risk, and Clusters 3 (38.5% AEs), 4 (60% AEs), and 5 (66.7% AEs) have high risk (*Figure 7*; *P* = 0.0002), even when corrected for age, sex, and NT-proBNP (*Table 2*).

**Table 2:** Cox regression for time to adverse event across the R14^Δ/+^ clusters

Unadjusted						
Cluster	HR	SE	Z-value	*P*-value	95% CI	
1	2.00e+09	1.33e+09	32.21	<0.001	5.44e+08	7.37e+09
2	Reference					
3	4.73e+09	3.52e+09	29.97	<0.001	1.10e+09	2.03e+10
4	7.73e+09					
5	1.54e+10	1.18e+10	30.41	<0.001	3.39e+09	6.97e+10

Adjusted for age, sex, and NT-proBNP						
Cluster	HR	SE	Z-value	*P*-value	95% CI	
1	1.31e+09	1.08e+09	25.58	<0.001	2.63e+08	6.57e+09
2	Reference					
3	2.02e+09	1.57e+09	27.58	<0.001	4.41e+08	9.28e+09
4	4.17e+09					
5	5.58e+09	4.43e+09	28.29	<0.001	1.18e+09	2.64e+10
Age	101.788	0.0198438	0.91	0.363	0.9797201	1.057.525
Gender	1.195.265	0.5938615	0.36	0.720	0.4513865	3.165.042
logNT-proBNP	1.274.732	0.2208389	1.40	0.161	0.9077242	1.790.127

The upper panel presents univariable Cox regression analyses for the association between Clusters 1 and 5 (*N* = 87 R14^Δ/+^ carriers) and the composite adverse clinical endpoint (HF hospitalization, device implantation and heart transplant/LVAD incidence, and all-cause mortality). The lower panel presents multivariable Cox regression analyses adjusted for age, sex, and NT-proBNP. Clusters 2 and 4 exhibited substantial differences in their characteristics, preventing meaningful comparative analysis using STATA. Likelihood ratio tests were used to assess statistical significance in Cox regression analyses.

CI, confidence interval; HR, hazard ratio; SE, standard error.

## 4. Discussion

Understanding the biological pathways underlying R14^Δ/+^ cardiomyopathy is essential for developing targeted therapeutic approaches. Circulating biomolecules, such as proteins, metabolites, and lipids, can provide insight into disease-related processes, including cardiac remodelling and fibrosis. For example, elevated plasma levels of extracellular matrix proteins or growth factors may reflect ongoing structural and functional changes in the heart.^8^ Unsupervised clustering of plasma proteomics revealed R14^Δ/+^ carrier subgroups with distinct molecular pathways associated with disease progression, offering new insights into the mechanisms driving R14^Δ/+^ cardiomyopathy.

Five clusters were identified across the R14^Δ/+^ disease spectrum. Clusters 1 and 2 are unaffected R14^Δ/+^ clusters based on NT-proBNP and LVEF, but with vastly different plasma proteome profiles, suggestive of distinct underlying compensatory mechanisms or early disease processes. In addition, Cluster 1 has elevated energy metabolism–related metabolites, which correlate with apoptosis-related proteins, which is also observed in the advanced disease Cluster 4. Therefore, Cluster 1 could be an R14^Δ/+^ cluster with a high metabolic demand, representing ongoing damage to the myocardium, at risk for disease development. Pathway enrichment analysis supports this by showing an enrichment of Rho GTPase-related pathways that are highly relevant in HF (i.e. cardiomyocyte function, hypertrophy, and fibrosis).^10^ The upregulation of inflammatory and cell cycle–related pathways, such as nucleotide-binding oligomerization domain (NOD)-like receptor, in plasma may indicate an attempt by cardiac cells to repair or remodel tissue.^11,12^ Clinical follow-up of the R14^Δ/+^ carriers included in this study confirmed that Cluster 1 is indeed the seemingly asymptomatic cluster at risk for R14^Δ/+^ cardiomyopathy progression as a higher cumulative incidence of AEs was observed. Clusters 3, 4, and 5 are symptomatic R14^Δ/+^ carriers with elevated NT-proBNP and reduced LVEF, that are characterized by elevated HF-related metabolites (i.e. SDMA). Here, Clusters 4 and 5 are end-stage R14^Δ/+^ clusters with vastly different plasma proteome profiles. Cluster 4, in line with Clusters 1 and 3, has a higher metabolic rate, as evidenced by the increase in circulating energy metabolism–related nucleotides. We postulate that this cluster represents ongoing damage to the myocardium, represented by the higher metabolic demand and increased apoptosis-related proteins. Pathway enrichment supports this indicating a heart under chronic strain, attempting to adapt to the progressive demands of HF. In addition, partial overlap in differentially expressed proteins between clusters suggests that the molecular pathways of R14^Δ/+^ cardiomyopathy develop progressively rather than in distinct stages. Proteins linked to apoptosis and cellular stress responses were upregulated in both Cluster 1 and Cluster 4, implying that pathways underlying myocardial injury and remodelling are active across disease clusters. This finding supports the concept that core molecular pathogenic processes are shared across the clinical spectrum of the disease. In contrast, Cluster 5 is another disease cluster with high NT-proBNP and low LVEF, but low energy metabolism–related metabolites. Our data suggest that this cluster represents a stable fibrotic end-stage of R14^Δ/+^, in which cardiac function is decreased.

SDMA, NAA, aconitic acid, SAMe, AICAR, and succinate were identified as metabolites that associate to R14^Δ/+^ disease state as they were identified to be elevated in R14^Δ/+^ disease clusters (*Table 3*). SDMA is a metabolite that reflects alterations in nitric oxide metabolism, oxidative stress and renal function, all of which play an important role in HF. SDMA is a metabolite that is known to associate with HF and worse hospital outcomes through altered LVEF.^13^ NAA is primarily a brain metabolite, but NAA synthesis is dependent on mitochondrial activity^14^; therefore, changes in NAA levels may indirectly reflect broader metabolic disturbances, including those occurring in HF, allowing NAA to be a novel metabolite associated to HF. Aconitic acid is an intermediate in the tricarboxylic acid (TCA) cycle, which is critical for energy metabolism.^15^ There have been no studies to date linking aconitic acid levels to HF outcomes. However, given its role in mitochondrial energy metabolism and sensitivity to oxidative stress,^15^ aconitic acid may serve as a metabolite that is indirectly associated to HF pathophysiology. SAMe is an important methyl donor in cellular metabolism.^16^ Elevated levels of SAMe are not directly associated with HF, but SAMe metabolism is indirectly involved in HF pathophysiology via its association with epigenetic changes (methylation), oxidative stress, and inflammation.^16^ AICAR is an intermediate in the purine biosynthesis pathway and an activator of adenosine monophosphate (AMP)-activated protein kinase. AICAR has been implicated in HF, due to its role in cellular energy metabolism. Elevated AICAR levels induce an adaptive response to maintain energy balance by stimulating adenosine monophosphate-activated protein kinase (AMPK), which increases glucose uptake, fatty acid oxidation, and mitochondrial biogenesis,^17^ which may be protective in HF. Succinate is an intermediate in the TCA cycle. Elevated succinate levels have previously been associated with HF, as its accumulation occurs due to impaired succinate dehydrogenase activity, reflecting a metabolic shift away from efficient oxidative phosphorylation towards less efficient energy production pathways such as glycolysis.^22^ Six metabolites were identified to associate to different R14^Δ/+^ carrier clusters, of which SDMA, AICAR, and succinate are known to be associated with HF. In contrast, NAA, aconitic acid, and SAMe are novel metabolites associated to HF that can indirectly be related to HF pathophysiology.

**Table 3.** Circulating metabolites associating to R14^Δ/+^ clusters

Metabolite	Role	Association with HF	Known?	Ref
Circulating metabolites				
SDMA	An endogenous inhibitor of nitric oxide synthase, impairing nitric oxide (NO) production.	Elevated SDMA levels are associated with impaired vascular function, oxidative stress, and renal dysfunction, which is common in HF.	Yes	13
NAA	A marker of neuronal health and function, and its presence in cardiac contexts unknown.	Altered energy metabolism and mitochondrial dysfunction in HF might influence NAA levels indirectly through systemic changes.	No	14
Cis-aconitic acid	An intermediate in TCA cycle, and levels can indicate mitochondrial dysfunction, a hallmark of HF.	Accumulation of TCA intermediates like cis-aconitic acid could reflect disrupted energy metabolism in failing myocardium.	No	15
SAMe	A key methyl donor in cellular metabolism and epigenetic regulation.	In HF, disruptions in methionine metabolism and methylation pathways could contribute to disease progression. In addition, SAMe has roles in oxidative stress response and inflammation.	No	16
AICAR	An activator of AMPK, a key regulator of energy balance.	In HF, AMPK activation may be protective by enhancing glucose uptake, fatty acid oxidation, and mitochondrial biogenesis. Elevated AICAR could reflect compensatory mechanisms in response to energy deficits in HF.	Yes	17
Succinate	A TCA cycle intermediate. Also acts as a signalling molecule in inflammation and oxidative stress.	Elevated succinate levels are linked to mitochondrial dysfunction, a key feature in HF.	Yes	22
Circulating nucleotide-derived metabolites				
GDP-mannose	A nucleotide sugar involved in glycosylation, specifically in the biosynthesis of glycoproteins and glycolipids.	Elevation could reflect changes in glycosylation, which is a key feature of cardiac remodelling in HF, linked to myocardial fibrosis and structural changes, which are central to HF progression.	No	19
ATP	The primary energy currency of the cell and is crucial for myocardial contraction and relaxation.	ATP depletion is a hallmark of HF due to mitochondrial dysfunction and impaired oxidative phosphorylation. Low ATP levels contribute to reduced cardiac output in HF.	Yes	20,21,27
ADP-ribose	Involved in signalling pathways, such as poly (ADP-ribose) polymerase (PARP) activation, which regulates DNA repair and stress responses.	Elevated ADP-ribose levels reflect PARP activation, a response to oxidative stress and DNA damage, which are prominent in HF. PARP-driven NAD+ consumption and cell death are critical processes in HF.	Yes	20,21,27
UDP-galactose	A nucleotide sugar involved in glycosylation and carbohydrate metabolism.	Elevated levels may indicate increased glycosylation of ECM proteins during remodelling. ECM changes are common in HF, contributing to fibrosis and stiffening of the heart.	No	19
UDP-glucose	Essential for glycogen synthesis and serves as a substrate for glycosylation.	Involved in both glycogen synthesis and glycosylation. Altered glucose metabolism and ECM remodelling are features of HF.	No	19
ADP	A precursor to ATP and plays a role in energy metabolism.	ADP accumulation occurs when ATP levels are low, signalling energy deficits in the heart. High ADP levels may reflect impaired mitochondrial function and energy metabolism issues, common in HF.	Yes	20,21,27
UDP	A precursor for nucleotide sugars and participates in glycosylation processes.	Involved in glycosylation and ECM remodelling. Elevated UDP levels may indicate altered metabolism and tissue remodelling in HF.	No	19
CDP-ethanolamine	Involved in the biosynthesis of phosphatidylethanolamine, a key phospholipid in cell membranes.	Involved in the synthesis of cell membrane components. Altered membrane composition and lipid remodelling are common in HF, particularly in response to mitochondrial dysfunction and myocardial injury.	No	19
UDP-glucA	A key cofactor in glucuronidation, involved in detoxification and glycosylation.	Involved in the synthesis of glycosaminoglycans, which are important in ECM remodelling and fibrosis in the heart.	No	19
Nicotinamide	Plays a role in various cellular processes (i.e. energy metabolism, DNA repair, and cellular signalling), primarily through its conversion into NAD+.	Crucial for mitochondrial function, energy metabolism, and cellular repair. NAD+ depletion is a hallmark of HF, and nicotinamide levels may indicate NAD+ deficits and mitochondrial stress.	Yes	20,21,27

Overview of circulating metabolites and nucleotide-derived metabolites identified as differentially abundant across R14^Δ/+^ carriers. The table summarizes the biological role of each metabolite and its known or proposed association with HF based on existing literature.

Energy metabolism–related metabolites were found to be altered across the R14^Δ/+^ disease spectrum, reflecting increased metabolic demands (in Clusters 1 and 4), consistent with the demands of tissue repair processes (*Table 3*). Increased glucose levels might indicate increased glycolysis and cellular respiration to meet the energy demands of cell growth, division, and tissue repair. This is further supported by the increased levels of ATP and ADP, as they are direct indicators of high energy turnover and utilization within tissue repair.^23,24^ This is supported by an increase in apoptosis-related proteins, which reflect cell death and release of molecules into the circulation.^25^ In contrast, the lower levels of these metabolites in the other cluster (Clusters 2 and 5) suggest a reduced metabolic rate, which associates with different cellular functions such as maintenance or immune responses rather than tissue repair.^18,26^ GDP-mannose, UDP-galactose, UDP-glucose, and CDP-ethanolamine are nucleotide derivatives. ATP, ADP, and UDP are canonical nucleotides. ADP-ribose is nucleotide derivative, UDP-glucA is a derivative of nucleotide sugars, and nicotinamide is a precursor of NAD+.^19^ ATP, ADP, ADP-ribose, and nicotinamide are known to be strongly associated with HF,^20,21, 27^ whereas GDP-mannose, UDP-galactose, UDP-glucose, UDP, CDP, ethanolamine, and UDP-glucA are indirectly associated with HF through their involvement in energy metabolism, glycosylation, extracellular matrix (ECM) remodelling and oxidative stress (*Table 3*). Elevated levels may indicate impaired metabolic or structural pathways contributing to the disease process.

PC(16:0/22:2), PC(18:0/22:2), and LPE(18:2) are lipid species that are associated with the highly metabolically active disease Cluster 4. PCs are major components of cell membranes and influence membrane fluidity, signalling, and lipid metabolism,^28^ further supporting the suggestion that Cluster 4 is a cluster representing ongoing damage, by leakage of cell membrane components into the circulation. Interestingly, the increase is only observed in Cluster 4. To date, increased PC (16:0/22:2) and PC(18:0/22:2) have not been described in relation to HF. However, reduced LPE(18:2) levels have recently been associated with HF,^29^ which is in line with our data. The role of PC(16:0/22:2), PC(18:0/22:2), and LPE(18:2) in HF is limited. However, their involvement in lipid metabolism, oxidative stress, and inflammation suggests a link to tissue damage and HF pathophysiology.^28,30^

## 5. Limitations

Our study provides novel insights into the molecular pathways underlying R14^Δ/+^ cardiomyopathy and HF. However, several limitations should be acknowledged. First, longitudinal follow-up of plasma proteins is necessary to determine how these molecular changes evolve over time and their role in disease progression. In addition, modulation of these pathways in an experimental setting or intervention study would be required to prove causality. Without such data, we cannot yet establish whether these pathways contribute to clinical outcomes and/or predict R14^Δ/+^ cardiomyopathy onset and progression. Second, our study cohort was relatively small, reflecting the rarity of the R14^Δ/+^ pathogenic variant and the limited size of the affected disease population. Although the small cohort size may limit the generalizability of our findings, it is important to note that focusing on a specific and rare genetic aetiology provides a unique opportunity to explore disease mechanisms in a well-defined population. Future studies with larger sample sizes and replication in broader cohorts will be crucial to validate these findings and explore their applicability to HF and R14^Δ/+^ cardiomyopathy.

## 6. Conclusion

Unsupervised clustering of plasma proteomics revealed distinct molecular pathways associated with R14^Δ/+^ cardiomyopathy progression. Notably, energy-related metabolites were strongly associated with apoptosis-related proteins and associated with an increased risk to cardiac events in a seemingly asymptomatic R14^Δ/+^ cluster with low NT-proBNP and normal LVEF. These findings provide insight into the early molecular mechanisms driving R14^Δ/+^ cardiomyopathy and highlight potential pathways involved in disease progression. Further follow-up studies are needed to determine the clinical relevance of these molecular changes and their role in shaping future therapeutic strategies. In conclusion, we show that stratifying a cohort of R14^Δ/+^ carriers across the disease spectrum at a molecular level offers a promising tool for the detection of early mechanisms of disease onset and thereby potent targets for future therapies.

## Supplementary Material

cvag089_Supplementary_Data

## Data Availability

The data underlying this article are available from the corresponding author upon reasonable request.
