# Supplementary material for: Plasma proteomics stratification identifies phospholamban R14del carriers at risk for disease progression
Source: Cardiovasc Res. 2026 Apr 25;122(8):1104–18. doi: 10.1093/cvr/cvag089 (PMC13241056; doi:10.1093/cvr/cvag089)
Supplement: cvag089_Supplementary_Data [file cvag089_supplementary_data.zip › V2 Supp. Table 1. Total Baseline Characteristics.docx]

| **Factor** | **cluster = 1** | **cluster = 2** | **cluster = 3** | **cluster = 4** | **cluster = 5** | **p-value** | **Test** |
| --- | --- | --- | --- | --- | --- | --- | --- |
| N | 50 | 13 | 13 | 5 | 6 |  |  |
| Age, mean (SD) | 54.62 (13.51) | 48.46 (14.88) | 61.62 (12.19) | 45.60 (17.17) | 58.00 (9.94) | 0.078 | ANOVA |
| Sex (male/female) | 28 (56%) | 9 (69%) | 3 (23%) | 1 (20%) | 2 (33%) | 0.064 | Pearson’s chi-squared |
| BMI, mean (SD) | 26.17 (4.26) | 26.13 (2.08) | 26.51 (3.37) | 30.30 (6.81) | 25.10 (5.59) | 0.28 | ANOVA |
| Pulse, mean (SD) | 67.55 (13.04) | 70.50 (13.85) | 65.33 (10.00) | 80.20 (19.51) | 76.50 (15.15) | 0.14 | ANOVA |
| Systolic BP, mean (SD) | 124.46 (16.45) | 127.92 (16.50) | 113.50 (20.48) | 102.80 (16.18) | 104.50 (22.97) | 0.004 | ANOVA |
| Diastolic BP, mean (SD) | 76.80 (9.17) | 78.54 (8.84) | 72.08 (8.67) | 71.80 (11.52) | 64.50 (9.65) | 0.014 | ANOVA |
| NYHA class |  |  |  |  |  | <0.001 | Pearson’s chi-squared |
| No Heart failure | 38 (76%) | 10 (77%) | 2 (15%) | 1 (20%) | 0 (0%) |  |  |
| I | 3 (6%) | 1 (8%) | 2 (15%) | 0 (0%) | 0 (0%) |  |  |
| II | 8 (16%) | 1 (8%) | 6 (46%) | 1 (20%) | 1 (17%) |  |  |
| III | 1 (2%) | 1 (8%) | 3 (23%) | 0 (0%) | 3 (50%) |  |  |
| IV | 0 (0%) | 0 (0%) | 0 (0%) | 3 (60%) | 2 (33%) |  |  |
| Previous HF hospitalization | 3 (6%) | 0 (0%) | 4 (31%) | 2 (40%) | 4 (67%) | <0.001 | Pearson’s chi-squared |
| **Laboratory** |  |  |  |  |  |  |  |
| N | 50 | 13 | 13 | 5 | 6 |  |  |
| NTproBNP (ng/L), median (IQR) | 179 (93, 444) | 57 (10, 111) | 1101 (495, 2313) | 3469 (932, 4263) | 2852 (762, 9344) | <0.001 | Kruskal-Wallis |
| cTNT (ng/L), median (IQR) | 11 (8, 18) | 12 (7, 13) | 20 (13, 39) | 23 (16, 24) | 22 (18, 34) | 0.001 | Kruskal-Wallis |
| **Echocardiograph** |  |  |  |  |  |  |  |
| N | 30 | 11 | 10 | 4 | 6 |  |  |
| LVEF, mean (SD) | 47.52 (13.00) | 49.03 (13.37) | 31.50 (12.36) | 17.50 (11.90) | 25.17 (10.76) | <0.001 | ANOVA |
| LVEDd, mean (SD) | 51.21 (6.69) | 51.35 (5.47) | 52.20 (7.30) | 58.30 (5.53) | 57.03 (7.57) | 0.13 | ANOVA |
| LVEDs, mean (SD) | 37.12 (10.23) | 37.28 (9.47) | 44.30 (7.35) | 49.67 (8.08) | 51.17 (7.87) | 0.004 | ANOVA |
| GLS, mean (SD) | -15.71 (3.49) | -16.70 (2.86) | -12.18 (3.91) | . (.) | -13.45 (7.14) | 0.22 | ANOVA |
| RV peak pressure, mean (SD) | 20.70 (8.41) | 17.65 (11.29) | 28.40 (9.25) | 13.17 (10.38) | 25.85 (6.85) | 0.088 | ANOVA |
| TAPSE, mean (SD) | 21.70 (4.85) | 22.55 (2.38) | 16.64 (3.47) | 13.95 (3.82) | 18.80 (4.62) | <0.001 | ANOVA |
| **ECG** |  |  |  |  |  |  |  |
| N | 50 | 13 | 13 | 5 | 6 |  |  |
| Rhythm |  |  |  |  |  | 0.13 | Pearson’s chi-squared |
| Sinus | 49 (98%) | 13 (100%) | 11 (85%) | 4 (80%) | 6 (100%) |  |  |
| Atrial fibrillation | 0 (0%) | 0 (0%) | 1 (8%) | 1 (20%) | 0 (0%) |  |  |
| Paced | 1 (2%) | 0 (0%) | 1 (8%) | 0 (0%) | 0 (0%) |  |  |

**Supplementary Table 1. Total baseline characteristics across the R14^Δ/+^ clusters.**
