# Supplementary material for: Plasma proteomics stratification identifies phospholamban R14del carriers at risk for disease progression
Source: Cardiovasc Res. 2026 Apr 25;122(8):1104–18. doi: 10.1093/cvr/cvag089 (PMC13241056; doi:10.1093/cvr/cvag089)
Supplement: cvag089_Supplementary_Data [file cvag089_supplementary_data.zip › V2 Supp. Table 2. Comorbidities.docx]

**Supplementary Table 2. Comorbidities across the R14^Δ/+^ clusters.**

| **Clusters** |  | **Overall** | **Cluster 1** | **Cluster 2** | **Cluster 3** | **Cluster 4** | **Cluster 5** | **p-value*** |
| --- | --- | --- | --- | --- | --- | --- | --- | --- |
|  | N | 87 | 50 | 13 | 13 | 5 | 6 |  |
| Hypertension | 87 | 5,7% (5/87) | 6,0% (3/50) | 0% (0/13) | 7,7% (1/13) | 20% (1/5) | 0% (0/6) | 0.5 |
| Hypercholesterolemia | 87 | 4,6% (4/87) | 4,0% (2/50) | 7,7% (1/13) | 0% (0/13) | 20% (1/5) | 0% (0/6) | 0.3 |
| Diabetes | 87 | 2,3% (2/87) | 2,0% (1/50) | 0% (0/13) | 0% (0/13) | 0% (0/5) | 17% (1/6) | 0.3 |
| MI | 87 | 4,6% (4/87) | 6,0% (3/50) | 7,7% (1/13) | 0% (0/13) | 0% (0/5) | 0% (0/6) | >0.9 |
| AF | 87 | 18% (16/87) | 18% (9/50) | 0% (0/13) | 23% (3/13) | 20% (1/5) | 50% (3/6) | 0.094 |
| Kidney Disease | 87 | 4,6% (4/87) | 0% (0/50) | 0% (0/13) | 31% (4/13) | 0% (0/5) | 0% (0/6) | 0.002 |

* Fisher’s Exact Test for Count Data with simulated p-value (based on 2000 replicates)

AF, atrial fibrillation; MI, myocardial infarction;
