# Supplementary material for: Plasma proteomics stratification identifies phospholamban R14del carriers at risk for disease progression
Source: Cardiovasc Res. 2026 Apr 25;122(8):1104–18. doi: 10.1093/cvr/cvag089 (PMC13241056; doi:10.1093/cvr/cvag089)
Supplement: cvag089_Supplementary_Data [file cvag089_supplementary_data.zip › V2 Supp. Table 3. Medication.docx]

**Supplementary Table 3. Medication usage across the R14^Δ/+^ clusters.**

| **Clusters** |  | **Overall** | **Cluster 1** | **Cluster 2** | **Cluster 3** | **Cluster 4** | **Cluster 5** | **p-value*** |
| --- | --- | --- | --- | --- | --- | --- | --- | --- |
|  | N | 87 | 50 | 13 | 13 | 5 | 6 |  |
| Medication | 87 | 79% (69/87) | 76% (38/50) | 62% (8/13) | 92% (12/13) | 100% (5/5) | 100% (6/6) | 0.2 |
| Beta blocker | 87 | 64% (56/87) | 64% (32/50) | 46% (6/13) | 69% (9/13) | 80% (4/5) | 83% (5/6) | 0.8 |
| Diuretics | 87 | 32% (28/87) | 22% (11/50) | 0% (0/13) | 69% (9/13) | 60% (3/5) | 83% (5/6) | <0.001 |
| ACE/ARB | 87 | 39% (34/87) | 50% (25/50) | 31% (4/13) | 15% (2/13) | 20% (1/5) | 33% (2/6) | 0.14 |
| MRA | 87 | 33% (29/87) | 20% (10/50) | 7.7% (1/13) | 62% (8/13) | 80% (4/5) | 100% (6/6) | <0.001 |
| ARNI | 87 | 20% (17/87) | 10% (5/50) | 7.7% (1/13) | 54% (7/13) | 20% (1/5) | 50% (3/6) | 0.002 |
| SGLT2i | 87 | 17% (15/87) | 10% (5/50) | 7.7% (1/13) | 15% (2/13) | 60% (3/5) | 67% (4/6) | 0.001 |
| OAC | 87 | 13% (11/87) | 8.0% (4/50) | 0% (0/13) | 23% (3/13) | 20% (1/5) | 50% (3/6) | 0.023 |
| Anti-platelet | 87 | 5.7% (5/87) | 6.0% (3/50) | 7.7% (1/13) | 7.7% (1/13) | 0% (0/5) | 0% (0/0) | >0.9 |
| Inotropic | 87 | 8.0% (7/87) | 0% (0/50) | 0% (0/13) | 15% (2/13) | 60% (3/5) | 33% (2/6) | <0.001 |

* Fisher’s Exact Test for Count Data with simulated p-value (based on 2000 replicates)
