# Supplementary material for: Plasma proteomics stratification identifies phospholamban R14del carriers at risk for disease progression
Source: Cardiovasc Res. 2026 Apr 25;122(8):1104–18. doi: 10.1093/cvr/cvag089 (PMC13241056; doi:10.1093/cvr/cvag089)
Supplement: cvag089_Supplementary_Data [file cvag089_supplementary_data.zip › V2 Supp. Table 4. Comparison cluster vs medication.docx]

| **Effect Type** | **Mean R² (%)** | **Median R² (%)** | **Proteins >5% Effect** | **% of Total Proteins** |
| --- | --- | --- | --- | --- |
| **Disease Clusters** | 28.0 | 25.3 | 2375 | 91.0 |
| **Medication Burden** | 2.9 | 1.3 | 450 | 17.2 |
| **Both Combined** | 29.4 | 26.7 | 2452 | 93.9 |

**Supplementary Table 4.** **Comparison of disease cluster versus medication burden effects on protein expression.**
