# Supplementary material for: Plasma proteomics stratification identifies phospholamban R14del carriers at risk for disease progression
Source: Cardiovasc Res. 2026 Apr 25;122(8):1104–18. doi: 10.1093/cvr/cvag089 (PMC13241056; doi:10.1093/cvr/cvag089)
Supplement: cvag089_Supplementary_Data [file cvag089_supplementary_data.zip › V2 Supp. Table 5. Unsupervised Clustering.docx]

**Supplementary Table 5.** **Validation of medication-sensitive protein identification.**

| **Protein Status** | **Number of Proteins** | **Mean Medication R² (%)** | **Mean Cluster R² (%)** | **% with >5% Medication Effect** |
| --- | --- | --- | --- | --- |
| **Retained** | 2100 | 1.2 | 27.4 | 0.0 |
| **Removed (Medication-Sensitive)** | 511 | 9.8 | 30.6 | 88.1 |
