# Supplementary material for: Plasma proteomics stratification identifies phospholamban R14del carriers at risk for disease progression
Source: Cardiovasc Res. 2026 Apr 25;122(8):1104–18. doi: 10.1093/cvr/cvag089 (PMC13241056; doi:10.1093/cvr/cvag089)
Supplement: cvag089_Supplementary_Data [file cvag089_supplementary_data.zip › V2 Supp. Table 6. Metabolite list.docx]

**Supplementary Table 4. List of metabolites measured across R14^Δ/+^ clusters**

| 1-Methyladenosine | ADP Ribose | Creatinine | Flavine-adenine-dinucleotide |
| --- | --- | --- | --- |
| 1-Methylnicotinamide | Agmatine | Crotonyl CoA | Fructose-1,6-bisphosphatase |
| 2-3-cGMP AMP | AICAR | Cystathionine | Ferrulic acid |
| 2-Aminoadipic acid | AICAR-P | Cysteine | Folate |
| 2-Deoxyadenosine | Aketoglutaric acid | Cysteine sulfate | Fructose-1-P |
| 2-Methyl-Butyrylcarnitine | Alanine | Cysteine sulfinic acid | Fructose-6-P |
| 3-3-cGMP AMP | Alanylglutamine | Cystine | Fumarate |
| 3-Aminobutyric acid | Allantoin | Cytidine | Glycerol-3-P |
| 3-Methylcytidine | AMP | Cytosine | Galactose-1-P |
| 3-Methyltetrahydrofuran | Anserine | dADP | Glyceraldehyde 3-P |
| 4-Hydroxyproline | Anthanillic acid | dAMP | Guanylate-binding protein-1 |
| 5-Hydroxylysine | Arginine | dATP | GDP |
| 7-Methylxanthine | Argininosuccinate | dCDP | GDP fucose |
| 8-OH-2-deoxyguanosine | Asparagine | dCMP | GDP mannose |
| AC(0:0)Carnitine | Aspartic acid | dCTP | Gluconic acid |
| AC(10:0) | ATP | Decanoic acid | Glucosamine-6-P |
| AC(12:0) | Barbituric acid | d-carnitine | Glucose-1-P |
| AC(14:0) | Benzoic acid | d-cytidine | Glucose-6-P |
| AC(16:0) | Betaine | d-guanosine | Glucuronic acid |
| AC(16:1) | Butyryl CoA | d-inosine | Glutamate |
| AC(18:0) | cAMP | d-uridine | Glutamic acid |
| AC(18:1) | cCMP | dGMP | Glutamine |
| AC(18:2) | c-di-AMP | Dihydrofolic acid | Glutamylalanine |
| AC(18:3) | c-di-GMP | Dihydromethyluracil | Glutamylcysteine |
| AC(2:0) | CDP | Dihydroorotic acid | Glutaric acid |
| AC(3:0) | CDP Choline | Dihydrouracil | Glutathioneox |
| AC(4:0) | CDP Ethanolamine | Dihydroxy acetone phosphate | Glutathionered |
| AC(5:0) | cGMP | Dimethylglycine | Glycerophosphocholine |
| AC(6:0) | Choline | Dimethyllysine | Glycine |
| AC(8:0) | Cinnamic acid | D-inosine monophosphate | Glyoxyllic acid |
| Acetoacetyl CoA | Cis-aconitic acid | Dinitrosalicylic acid | GMP |
| Acetylcholine | Citramalic acid | Di-OH-phenylacetic acid | GTP |
| Acetyl CoA | Citrate | D-methylthioadenosine | Guanine |
| Acetylneuraminic acid | Citrulline | Dopamine | Guanosine |
| Adenine | CMP | D-pantothenic acid | Haxnoic acid |
| Adenosine | CMP acetylneuraminic acid | D-Ribose-5-P | Hydroxy indoleacetic acid |
| Adenylsuccinate | CoA | D-S-adenosylmethionine | Hippuric acid |
| Adipic acid | Corticosterone | dUMP | Histamine |
| ADMA | Coumaric acid | dUTP | Histidine |
| ADP | Creatine | Epinephrine | HMDC |

Table 4 – Continued

| Hydroxymethyluracil | Methyl Malonyl CoA | P-serine | Tryptophan |
| --- | --- | --- | --- |
| 3-Hydroxyphenylpropionic acid | Methylxanthine | Putrescine | TTP |
| hydroxyglutaric acid | Mevalonic acid | Pyridoxal | Tyramine |
| hydroxyisocaproic acid | N-acetyl aspartate | Pyridoxal-5-P | Tyrosine |
| hydroxymethylglutarylCoa | N-acetyl cysteine | Pyridoxine | UDP |
| hydroxyphenylaceticacid | N-acetyl glucosamine | Pyroglutamic acid | UDP-acetyl galactosamine |
| Hypotaurine | N-acetyl glutamic acid | Pyruvate | UDP-acetyl glucosamine |
| Hypoxanthine | NAD | Quinolinic acid | UDP-galactose |
| Indole-3-carboxaldehyde | NADH | Riboflavin | UDP-glucose |
| Inosine monophosphate | NADP | Riboflavin-5-P | UDP-glucuronic acid |
| Indole | NADPH | Ribose-5-P | UMP |
| Indole-3-propionic acid | Nicotinamide | Ribulose-5-P | Uracil |
| Indoleacetic acid | Nicotinamide mononucleotide | S-5-adenosyl-L-cysteine | Ureidopropionic acid |
| Indolelactic acid | Nicotinamide riboside | S-5-adenosyl-L-homocysteine | Ureidosuccinic acid |
| Inosine | Nicotinic acid | S-adenosyl-cysteine | Uridine |
| Isobutyryl carnitine | Nitrophenol | S-adenosyl-L-homocysteine | Urocanic acid |
| Isobutyryl CoA | Norepinephrine | S-adenosyl-L-methionine | Valeric acid |
| Isocitratesp | Octanoic acid | Salicylic acid | Valine |
| Isoleucine | OH-butyryl CoA | Sarcosine | Xanthine |
| Isovaleryl CoA | OH-phenyllactic acid | SDMA | Xanthosine |
| Itaconic acid | OH-proline | Serine | Xanthurenic acid |
| Kynurenic acid | Ophthalmic acid | Serotonine | Xylulose-5-P |
| Kynurenine | Ornithine | Shikimic acid |  |
| Lactate | Orotic acid | Sorbitol mannitol |  |
| Leucine | Oxalic acid | Spermidine |  |
| Lysine | Palmitic acid | Spermine |  |
| M2PY | Pantothenic acid | Succinate |  |
| M4PY | P-Creatine | Succinyl carnitine |  |
| Malate | Phenylalanine | Succinyl CoA |  |
| Malonic acid | Phenylethylamine | Taurine |  |
| Malonyl carnitine | Phenyl pyruvate | TDP |  |
| Malonyl CoA | Phosphocholine | Thiamine |  |
| Mannose-6-P | Phosphoenolpyruvate | Threonine |  |
| Melatonin | Phosphoethanolamine | Thymidine |  |
| Mercapturic acid | Phosphoglyceric acid | Thymine |  |
| Mesaconic acid | Phthalic acid | Thiamine pyrophosphate |  |
| Methionine | Pimelic acid | TMP |  |
| Methylarginine | Proline | Trimethylamine-N-oxide |  |
| Methylglutarylcarnine | Propionic acid | Trimethyllysine |  |
| Methyllysine | Propionyl CoA | Tryptamine |  |

AC, acyl-coenzyme a; AICAR, Acadesine; c, cyclic; CoA, coenzyme A; d-, deoxy; DP, diphosphate; MP, monophosphate; A, adenosine; TP, triphosphate; U, uridine; G, guanosine; T, thymidine; H, Hydroxide; OH, hydroxy; P-/-P, phospho/phosphate; SDMA, Symmetric Dimethylarginine; ADMA, Asymmetric dimethyl arginine; NAD, Nicotinamide-adenine-dinucleotide; HMDC, 3-Hydroxy-2-methyl-4-pyrone-6-carboxylic acid; M-PY, 1-methyl-pyridone-5-carboxamide
