# Supplementary material for: Plasma proteomics stratification identifies phospholamban R14del carriers at risk for disease progression
Source: Cardiovasc Res. 2026 Apr 25;122(8):1104–18. doi: 10.1093/cvr/cvag089 (PMC13241056; doi:10.1093/cvr/cvag089)
Supplement: cvag089_Supplementary_Data [file cvag089_supplementary_data.zip › V2 Supplemental Targeted Metabolomics TMET+ Method.docx]

**Supplementary Material**

**Supplementary Table and Figure Legends**

**Supplementary Table 1.** Total baseline characteristics across the R14Δ/+ clusters. Baseline characteristics of the R14Δ/+ carriers (N=87) stratified by R14Δ/+ clusters. N represents the number of individual patients per cluster. Data are presented as mean ± SD, median (IQR) or number (%), as indicated. P-values were calculated using one-way ANOVA, Kruskal-Wallis test, or Pearson’s chi-squared test, as appropriate.

**Supplementary Table 2.** Comorbidities across the R14Δ/+ clusters. Prevalence of comorbidities in R14Δ/+ carriers (N=87) stratified by R14Δ/+ clusters. N represents the number of individual patients per cluster. Data are presented as number (%). P-values were calculated using Fisher’s exact test.

**Supplementary Table 3.** Medication usage across the R14Δ/+ clusters. Medication use in R14Δ/+ carriers (N=87) stratified by R14Δ/+ clusters. N represents the number of individual patients per cluster. Data are presented as number (%). P-values were calculated using Fisher’s exact test.

**Supplementary Table 4.** Comparison of disease cluster versus medication burden effects on protein expression. Summary statistics comparing the relative contributions of cluster assignment versus medication burden to protein expression variance across all 2,611 proteins (in N=87 R14Δ/+ carriers). Partial R² values represent the percentage of variance uniquely explained by each factor using linear regression models. Disease clusters show substantially larger effects than medication burden across all metrics, with mean effects differing by an order of magnitude (28.0% vs 2.9%). The "Both Combined" row shows the total variance explained when both factors are included simultaneously in the model. The stark difference in proteins exceeding the 5% effect threshold (2,375 vs 450) demonstrates that disease biology dominates the proteomic signature, with medication effects representing a minority signal affecting fewer than one in five proteins.

**Supplementary Table 5.** Validation of medication-sensitive protein identification. Cross-validation analysis comparing proteins removed by delta AIC analysis (ΔAIC ≥2) versus those retained in clustering analysis. The table demonstrates that proteins identified as medication-sensitive by delta AIC criteria show significantly higher medication burden effects when assessed by partial R² analysis (9.8% vs 1.2% mean variance explained). The "% High Medication Effect" column shows the percentage of proteins in each group with >5% medication burden partial R², confirming that 88.1% of removed proteins had clinically meaningful medication effects while none of the retained proteins exceeded this threshold. The Adjusted Rand Index of the cluster stability after protein exclusion was 0.837. This validation confirms the concordance between our dual-metric approach and supports the biological relevance of the delta AIC selection criteria for identifying medication-confounded proteins.

**Supplementary Table 6.** List of metabolites measured across R14Δ/+ clusters. Comprehensive list of metabolites quantified in plasma samples from R14Δ/+ carriers included in the targeted metabolomics analyses.

**Supplementary Figure 1.** Visualization of outlier detection. Heatmap plotting protein expression across the R14Δ/+ carriers (N=88), derived from the Olink data with the outlier highlighted in green.

**Supplementary Figure 2.** Cluster number selection identifies five clusters as the optimal solution. A) Consensus clustering performed on proteomics data (N=87 R14Δ/+ carriers). Delta area analysis identifies that the optimal number of clusters could be six, however this splits cluster 2 into two very small subclusters, without increasing biological interpretability, therefore five clusters represents the optimal balance between biological interpretability and statistical robustness. B) Tracking plot uncovering how cluster composition would alter If consensus clustering were forced to identify 2-10 clusters. C) Consensus matrixes acquired when forcing consensus clustering to identify 3-6 clusters. Forcing 3 clusters shows that cluster 4 originates from cluster 1, while forcing 4 clusters show that cluster 5 originates from cluster 2. However, this results in loss of biological significance, as clusters 4 and 5 show an excellent stability score of 0.860 and 0.974 respectively, despite modest sample sizes.

**Supplementary Figure 3.** Pathway enrichment analysis of upregulated proteins of clusters across the R14Δ/+ spectrum. A) Cluster 1 enriched pathways based on 490 upregulated proteins. B) Cluster 2 enriched pathways based on 651 upregulated proteins. C) Cluster 4 enriched pathways based on 647 upregulated proteins. D) Cluster 5 enriched pathways based on 816 upregulated proteins. Analyses were based on plasma proteomics (N=87 R14Δ/+ carriers). Reactome database used for enrichment analysis with adjusted p-value <0.05.

**Supplementary Figure 4.** Pathway enrichment analysis of downregulated proteins of clusters across the R14Δ/+ spectrum. A) Cluster 1 enriched pathways based on 777 downregulated proteins, highlighting extracellular matrix, metabolism and inflammation. B) Cluster 2 enriched pathways based on 470 downregulated proteins, highlighting inflammation, extracellular matrix and metabolism. C) Cluster 4 enriched pathways based on 977 downregulated proteins, highlighting metabolism, apoptosis, stress and inflammation. D) Cluster 5 enriched pathways based on 508 downregulated proteins, highlighting apoptosis, autophagy, stress, inflammation, and metabolism. Analyses were based on plasma proteomics (N=87 R14Δ/+ carriers). Reactome database used for enrichment analysis with adjusted p-value <0.05.

**Targeted Metabolomics TMET+ Method**

**Summary**

A targeted metabolomics method was set up on the Waters Xevo TQS LC-MS system for plasma biomarkers. The method delivers semi-quantitative results in the form of peak area units (AU) or relative peak area units (RPA) when quantified versus the internal standard signal (RPA)

**Sample preparation**

This method is based on metabolite extraction from 20 µL plasma in NUNC-96 deep well plates, by adding 10 volumes (200 µL) of extraction solvent composed of ACN:MEOH:Water (40:40:20) The extraction solvent may contain stable isotope labelled internal standards for selected metabolites. The extract was mixed for 2 minutes and then centrifuged for 20 min at 4000 rpm at 4°C using a plate centrifuge prior to injection of 1 µL onto the LC-MS system

**LC Method - “TMET_HILIC_10min”**

Separation is achieved on a BEH Amide 2.1 x 100 mm column in HILIC mode using the “Generic HILIC” mobile phases. Column temperature is 30°C. The original method of 20 minutes has been compressed to 10 minutes with maintained separation of all included metabolites. Mobile phase A is 5 mM ammonium formate and 0.1% formic acid in 95% acetonitrile and 5% water. Mobile phase B is 10 mM ammonium formate and 0.1% formic acid in 100% water. The gradient is given in Table 1.

Table 1. LC-Gradient for Generic HILIC

Initial 0.400 100.0 0.0 Initial

1.00 0.400 100.0 0.0 6

7.00 0.400 45.0 55.0 6

8.00 0.400 45.0 55.0 6

8.10 0.400 100.0 0.0 6

11.00 0.400 100.0 0.0 6

**MS-MS Method – “TMET-2023-ESI+”**

The MS-MS method contains 85 MRM transitions covering about 65 unique metabolites, a few isomers to cover a wider concentration range for high abundant metabolites, and about 20 internal standards. The method is segmented in 5 sections (functions) due to a limitation of the software to 32 analytes per function, and due to a limitation of total number of analytes measured at the same time with a sufficiently long dwell time to allow for about 12 data points per peak. The MRM transitions and function are listed in Table 2.

Table 2. MRM transitions for TMET-2023-ESI+ “TMET+”


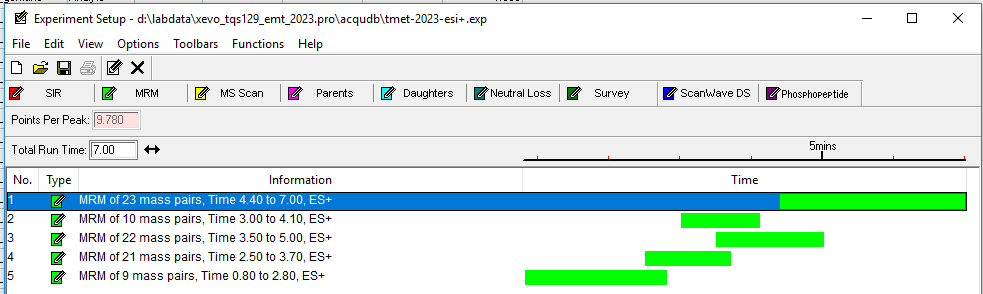


Function 5 0.8 – 2.0 min

| Nicotinamide | 123 | 80 | 1 | 0.006 | 35 | 20 |  | 0 | 1.31 |
| --- | --- | --- | --- | --- | --- | --- | --- | --- | --- |
| Nicotinamide-13C6 | 129 | 85 | 1 | 0.006 | 30 | 20 |  | 0 | 1.31 |
| M2PY | 153 | 108 | 1 | 0.006 | 35 | 25 |  | 0 | 1.69 |
| M4PY | 153 | 136 | 1 | 0.006 | 20 | 15 |  | 0 | 1.87 |
| M2PY P+1 D+1 | 154 | 109 | 1 | 0.006 | 35 | 25 |  | 0 | 1.69 |
| M4PY P+1 D+1 | 154 | 137 | 1 | 0.006 | 20 | 15 |  | 0 | 1.87 |
| M2PY-IS | 156 | 113 | 1 | 0.006 | 30 | 20 |  | 0 | 1.69 |
| Hippuric acid | 180 | 105 | 1 | 0.006 | 15 | 15 |  | 0 | 1.91 |
| D-pantothenic acid | 220 | 202 | 1 | 0.006 | 20 | 10 |  | 0 | 2.4 |

| Function 2 3.0 – 4.1 min |  |  |  |  |  |  |  |  |  |
| --- | --- | --- | --- | --- | --- | --- | --- | --- | --- |
| Trimethylamine N-oxide (TMAO) | 76 | 58 | 1 | 0.003 | 25 | 20 |  | 0 | 3.6 |
| 1-Methylnicotinamide | 137 | 94 | 1 | 0.003 | 25 | 20 |  | 0 | 3.81 |
| 1-MNA-D3 | 140 | 97 | 1 | 0.003 | 30 | 20 |  | 0 | 3.81 |
| Serotonin | 177 | 160 | 1 | 0.003 | 10 | 10 |  | 0 | 3.53 |
| Kynurenic acid | 190 | 144 | 1 | 0.003 | 25 | 20 |  | 0 | 3.6 |
| Kynurenic acid-D5 | 195 | 149 | 1 | 0.003 | 25 | 20 |  | 0 | 3.6 |
| AC(4:0) | 232 | 85 | 1 | 0.003 | 30 | 20 |  | 0 | 3.49 |
| AC(4:0) P+1 D+1 | 233 | 86 | 1 | 0.003 | 30 | 20 |  | 0 | 3.49 |
| AC(5:0) | 246 | 85 | 1 | 0.003 | 30 | 20 |  | 0 | 3.28 |
| AC(6:0) | 260 | 85 | 1 | 0.003 | 30 | 20 |  | 0 | 3.15 |

Function 4 2.5 – 3.7 min

| Choline | 104 | 60 | 1 | 0.003 | 25 | 15 |  | 0 | 3.42 |
| --- | --- | --- | --- | --- | --- | --- | --- | --- | --- |
| Choline P+1 D+1 | 105 | 61 | 1 | 0.003 | 25 | 15 |  | 0 | 3.42 |
| Choline-D9 | 113 | 69 | 1 | 0.003 | 25 | 15 |  | 0 | 3.42 |
| Creatinine | 114 | 86 | 1 | 0.003 | 25 | 10 |  | 0 | 3.37 |
| Creatinine P+1 D+1 | 115 | 87 | 1 | 0.003 | 25 | 10 |  | 0 | 3.37 |
| Creatinine-D3 | 117 | 89 | 1 | 0.003 | 20 | 10 |  | 0 | 3.37 |
| Hypoxanthine | 137 | 110 | 1 | 0.003 | 35 | 20 |  | 0 | 3.14 |
| 7-Methylxanthine | 167 | 124 | 1 | 0.003 | 25 | 20 |  | 0 | 2.7 |
| 2-Deoxyadenosine | 252 | 136 | 1 | 0.003 | 20 | 15 |  | 0 | 3.35 |
| Adenosine | 268 | 136 | 1 | 0.003 | 20 | 20 |  | 0 | 3.65 |
| AC(8:0) | 288 | 85 | 1 | 0.003 | 30 | 20 |  | 0 | 3 |
| AC(8:0) P+1 D+1 | 289 | 86 | 1 | 0.003 | 30 | 20 |  | 0 | 3 |
| AC(10:0) | 316 | 85 | 1 | 0.003 | 30 | 20 |  | 0 | 2.91 |
| AC(12:0) | 344 | 85 | 1 | 0.003 | 30 | 20 |  | 0 | 2.85 |
| AC(14:0) | 372 | 85 | 1 | 0.003 | 30 | 20 |  | 0 | 2.82 |
| AC(16:1) | 398 | 85 | 1 | 0.003 | 30 | 20 |  | 0 | 2.77 |
| AC(16:0) | 400 | 85 | 1 | 0.003 | 30 | 20 |  | 0 | 2.76 |
| AC(18:3) | 422 | 85 | 1 | 0.003 | 30 | 20 |  | 0 | 2.76 |
| AC(18:2) | 424 | 85 | 1 | 0.003 | 30 | 20 |  | 0 | 2.75 |
| AC(18:1) | 426 | 85 | 1 | 0.003 | 30 | 20 |  | 0 | 2.73 |
| AC(18:0) | 428 | 85 | 1 | 0.003 | 30 | 20 |  | 0 | 2.75 |

| \| Function 3 3-5-5.0 min \|  \|  \|  \|  \|  \|  \|  \|  \| \|  \| \| --- \| --- \| --- \| --- \| --- \| --- \| --- \| --- \| --- \| --- \| --- \| \| Alanine \| 90 \| 44 \| 1 \| 0.003 \| 15 \| 10 \|  \| 0 \| 4.7 \| \| \| Sarcosine \| 90.1 \| 44 \| 1 \| 0.003 \| 15 \| 10 \|  \| 0 \| 4.71 \| \| \| Proline \| 116 \| 70 \| 1 \| 0.003 \| 20 \| 15 \|  \| 0 \| 4.4 \| \| \| Valine \| 118.1 \| 72 \| 1 \| 0.003 \| 15 \| 10 \|  \| 0 \| 4.41 \| \| \| Betaine P+1 D+1 \| 119 \| 60 \| 1 \| 0.003 \| 30 \| 15 \|  \| 0 \| 4.26 \| \| \| Taurine \| 126 \| 44 \| 1 \| 0.003 \| 25 \| 20 \|  \| 0 \| 4.43 \| \| \| Leucine & iso-Leucine \| 132 \| 86 \| 1 \| 0.003 \| 15 \| 15 \|  \| 0 \| 4.07 & 4.19 \| \| \| Creatine \| 132 \| 90 \| 1 \| 0.003 \| 10 \| 10 \|  \| 0 \| 4.7 \| \| \| 4-hydroxyproline \| 132.1 \| 86 \| 1 \| 0.003 \| 20 \| 15 \|  \| 0 \| 4.41 \| \| \| Creatine P+1 D+1 \| 133 \| 91 \| 1 \| 0.003 \| 10 \| 10 \|  \| 0 \| 4.7 \| \| \| Deoxycarnitine \| 146 \| 87 \| 1 \| 0.003 \| 25 \| 15 \|  \| 0 \| 4.4 \| \| \| Methionine \| 150 \| 61 \| 1 \| 0.003 \| 25 \| 40 \|  \| 0 \| 4.28 \| \| \| Methionine-13C-D3 \| 154 \| 65 \| 1 \| 0.003 \| 20 \| 20 \|  \| 0 \| 4.28 \| \| \| AC(0:0) P+1 D+1 \| 163 \| 86 \| 1 \| 0.003 \| 30 \| 20 \|  \| 0 \| 4.48 \| \| \| Phenylalanine \| 166 \| 120 \| 1 \| 0.003 \| 15 \| 15 \|  \| 0 \| 3.98 \| \| \| AC(2:0) P+1 D+1 \| 205 \| 86 \| 1 \| 0.003 \| 30 \| 20 \|  \| 0 \| 4.21 \| \| \| Tryptophan \| 205 \| 188 \| 1 \| 0.003 \| 15 \| 10 \|  \| 0 \| 3.95 \| \| \| Kynurenine \| 209 \| 192 \| 1 \| 0.003 \| 15 \| 10 \|  \| 0 \| 3.98 \| \| \| Tryptophan-D5 \| 210 \| 192 \| 1 \| 0.003 \| 15 \| 10 \|  \| 0 \| 3.95 \| \| \| Kynurenine-D4 \| 213 \| 196 \| 1 \| 0.003 \| 15 \| 10 \|  \| 0 \| 3.98 \| \| \| AC(3:0) \| 218 \| 85 \| 1 \| 0.003 \| 30 \| 20 \|  \| 0 \| 3.87 \| \| \| AC(3:0) P+1 D+1 \| 219 \| 86 \| 1 \| 0.003 \| 30 \| 20 \|  \| 0 \| 3.87 \| \|  \| Function 1 4.4-7.0 min \|  \|  \|  \|  \|  \|  \|  \|  \|  \| \| --- \| --- \| --- \| --- \| --- \| --- \| --- \| --- \| --- \| --- \| \| Glycine \| 76 \| 30 \| 1 \| 0.003 \| 20 \| 10 \|  \| 0 \| 4.85 \| \| Serine \| 106 \| 60 \| 1 \| 0.003 \| 15 \| 10 \|  \| 0 \| 5 \| \| Threonine \| 120 \| 74 \| 1 \| 0.003 \| 15 \| 10 \|  \| 0 \| 4.8 \| \| Ornithine \| 133 \| 70 \| 1 \| 0.003 \| 15 \| 15 \|  \| 0 \| 5.6 \| \| Asparagine \| 133 \| 74 \| 1 \| 0.003 \| 10 \| 15 \|  \| 0 \| 5.04 \| \| Glutamine \| 147 \| 130 \| 1 \| 0.003 \| 15 \| 10 \|  \| 0 \| 4.96 \| \| Lysine \| 147.1 \| 130 \| 1 \| 0.003 \| 15 \| 10 \|  \| 0 \| 5.56 \| \| Glutamic acid \| 148 \| 84 \| 1 \| 0.003 \| 20 \| 15 \|  \| 0 \| 5.02 \| \| Histidine \| 156 \| 110 \| 1 \| 0.003 \| 20 \| 15 \|  \| 0 \| 5.45 \| \| 2-Aminoadipic acid \| 162 \| 98 \| 1 \| 0.003 \| 15 \| 15 \|  \| 0 \| 4.8 \| \| 5-hydroxylysine \| 163 \| 128 \| 1 \| 0.003 \| 15 \| 10 \|  \| 0 \| 5.78 \| \| Arginine \| 175 \| 70 \| 1 \| 0.003 \| 20 \| 20 \|  \| 0 \| 5.47 \| \| Arginine P+1 D+1 \| 176 \| 71 \| 1 \| 0.003 \| 20 \| 20 \|  \| 0 \| 5.47 \| \| Citrulline \| 176 \| 159 \| 1 \| 0.003 \| 15 \| 10 \|  \| 0 \| 5.07 \| \| Citrulline-D6 \| 182 \| 165 \| 1 \| 0.003 \| 15 \| 10 \|  \| 0 \| 5.07 \| \| Tyrosine \| 182.1 \| 165 \| 1 \| 0.003 \| 15 \| 10 \|  \| 0 \| 5.08 \| \| Arginine 13C6 15N5 \| 185 \| 75 \| 1 \| 0.003 \| 25 \| 25 \|  \| 0 \| 5.47 \| \| ADMA \| 203 \| 46 \| 1 \| 0.003 \| 25 \| 15 \|  \| 0 \| 5.17 \| \| SDMA \| 203.1 \| 172 \| 1 \| 0.003 \| 25 \| 15 \|  \| 0 \| 5.14 \| \| ADMA-D7 \| 210 \| 77 \| 1 \| 0.003 \| 30 \| 25 \|  \| 0 \| 5.17 \| \| Anserine \| 241 \| 170 \| 1 \| 0.003 \| 20 \| 20 \|  \| 0 \| 5.43 \| \| S-Adenosyl-L-Homocysteine \| 385 \| 136 \| 1 \| 0.003 \| 20 \| 20 \|  \| 0 \| 5.05 \| \| S-Adenosyl-L-Methionine \| 399 \| 136 \| 1 \| 0.003 \| 20 \| 25 \|  \| 0 \| 5.68 \|   **Quantification Method – “TMET_2023_ESI+”**  The quantification method (TMET_2023_ESI+.mdb) contains 101 analytes including the metabolites, alternative isomers and the internal standards. There is no calibration curve associated with the quantification method or the TMET method. Results are reported as Area Units (AU) and Relative Peak Areas (RPA) can be calculated in excel. |  |  |  |  |  |  |  |  |  |
| --- | --- | --- | --- | --- | --- | --- | --- | --- | --- | --- | --- | --- | --- | --- | --- | --- | --- | --- | --- | --- | --- | --- | --- | --- | --- | --- | --- | --- | --- | --- | --- | --- | --- | --- | --- | --- | --- | --- | --- | --- | --- | --- | --- | --- | --- | --- | --- | --- | --- | --- | --- | --- | --- | --- | --- | --- | --- | --- | --- | --- | --- | --- | --- | --- | --- | --- | --- | --- | --- | --- | --- | --- | --- | --- | --- | --- | --- | --- | --- | --- | --- | --- | --- | --- | --- | --- | --- | --- | --- | --- | --- | --- | --- | --- | --- | --- | --- | --- | --- | --- | --- | --- | --- | --- | --- | --- | --- | --- | --- | --- | --- | --- | --- | --- | --- | --- | --- | --- | --- | --- | --- | --- | --- | --- | --- | --- | --- | --- | --- | --- | --- | --- | --- | --- | --- | --- | --- | --- | --- | --- | --- | --- | --- | --- | --- | --- | --- | --- | --- | --- | --- | --- | --- | --- | --- | --- | --- | --- | --- | --- | --- | --- | --- | --- | --- | --- | --- | --- | --- | --- | --- | --- | --- | --- | --- | --- | --- | --- | --- | --- | --- | --- | --- | --- | --- | --- | --- | --- | --- | --- | --- | --- | --- | --- | --- | --- | --- | --- | --- | --- | --- | --- | --- | --- | --- | --- | --- | --- | --- | --- | --- | --- | --- | --- | --- | --- | --- | --- | --- | --- | --- | --- | --- | --- | --- | --- | --- | --- | --- | --- | --- | --- | --- | --- | --- | --- | --- | --- | --- | --- | --- | --- | --- | --- | --- | --- | --- | --- | --- | --- | --- | --- | --- | --- | --- | --- | --- | --- | --- | --- | --- | --- | --- | --- | --- | --- | --- | --- | --- | --- | --- | --- | --- | --- | --- | --- | --- | --- | --- | --- | --- | --- | --- | --- | --- | --- | --- | --- | --- | --- | --- | --- | --- | --- | --- | --- | --- | --- | --- | --- | --- | --- | --- | --- | --- | --- | --- | --- | --- | --- | --- | --- | --- | --- | --- | --- | --- | --- | --- | --- | --- | --- | --- | --- | --- | --- | --- | --- | --- | --- | --- | --- | --- | --- | --- | --- | --- | --- | --- | --- | --- | --- | --- | --- | --- | --- | --- | --- | --- | --- | --- | --- | --- | --- | --- | --- | --- | --- | --- | --- | --- | --- | --- | --- | --- | --- | --- | --- | --- | --- | --- | --- | --- | --- | --- | --- | --- | --- | --- | --- | --- | --- | --- | --- | --- | --- | --- | --- | --- | --- | --- | --- | --- | --- | --- | --- | --- | --- | --- | --- | --- | --- | --- | --- | --- | --- | --- | --- | --- | --- | --- | --- | --- | --- | --- | --- | --- | --- | --- | --- | --- | --- | --- | --- | --- | --- | --- | --- | --- | --- | --- | --- | --- | --- | --- | --- | --- | --- | --- | --- | --- | --- | --- | --- | --- | --- | --- | --- | --- | --- | --- | --- | --- | --- | --- | --- | --- | --- | --- | --- | --- | --- | --- | --- | --- | --- | --- | --- | --- | --- | --- | --- | --- | --- | --- | --- | --- | --- | --- | --- | --- | --- | --- | --- | --- | --- | --- | --- | --- | --- | --- | --- | --- | --- | --- | --- | --- | --- | --- | --- | --- | --- |
|  |  |  |  |  |  |  |  |  |  |
|  |  |  |  |  |  |  |  |  |  |
|  |  |  |  |  |  |  |  |  |  |
|  |  |  |  |  |  |  |  |  |  |
|  |  |  |  |  |  |  |  |  |  |
|  |  |  |  |  |  |  |  |  |  |
