# Supplementary figures and images for: Plasma proteomics stratification identifies phospholamban R14del carriers at risk for disease progression
Source: Cardiovasc Res. 2026 Apr 25;122(8):1104–18. doi: 10.1093/cvr/cvag089 (PMC13241056; doi:10.1093/cvr/cvag089)

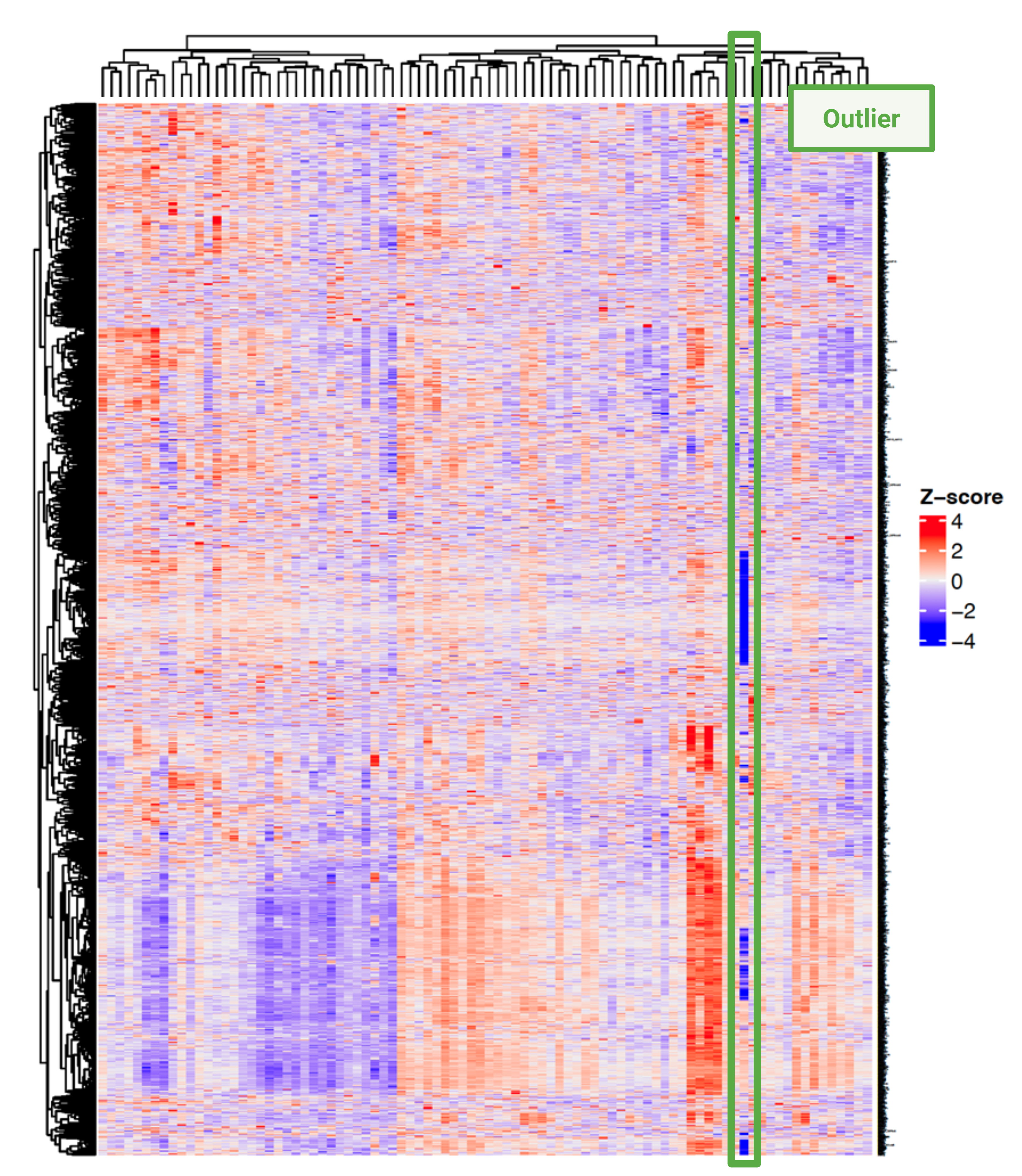

Supplement: cvag089_Supplementary_Data [file cvag089_supplementary_data.zip › V2 Sup Figure 1. Unsup Clustering.jpeg]

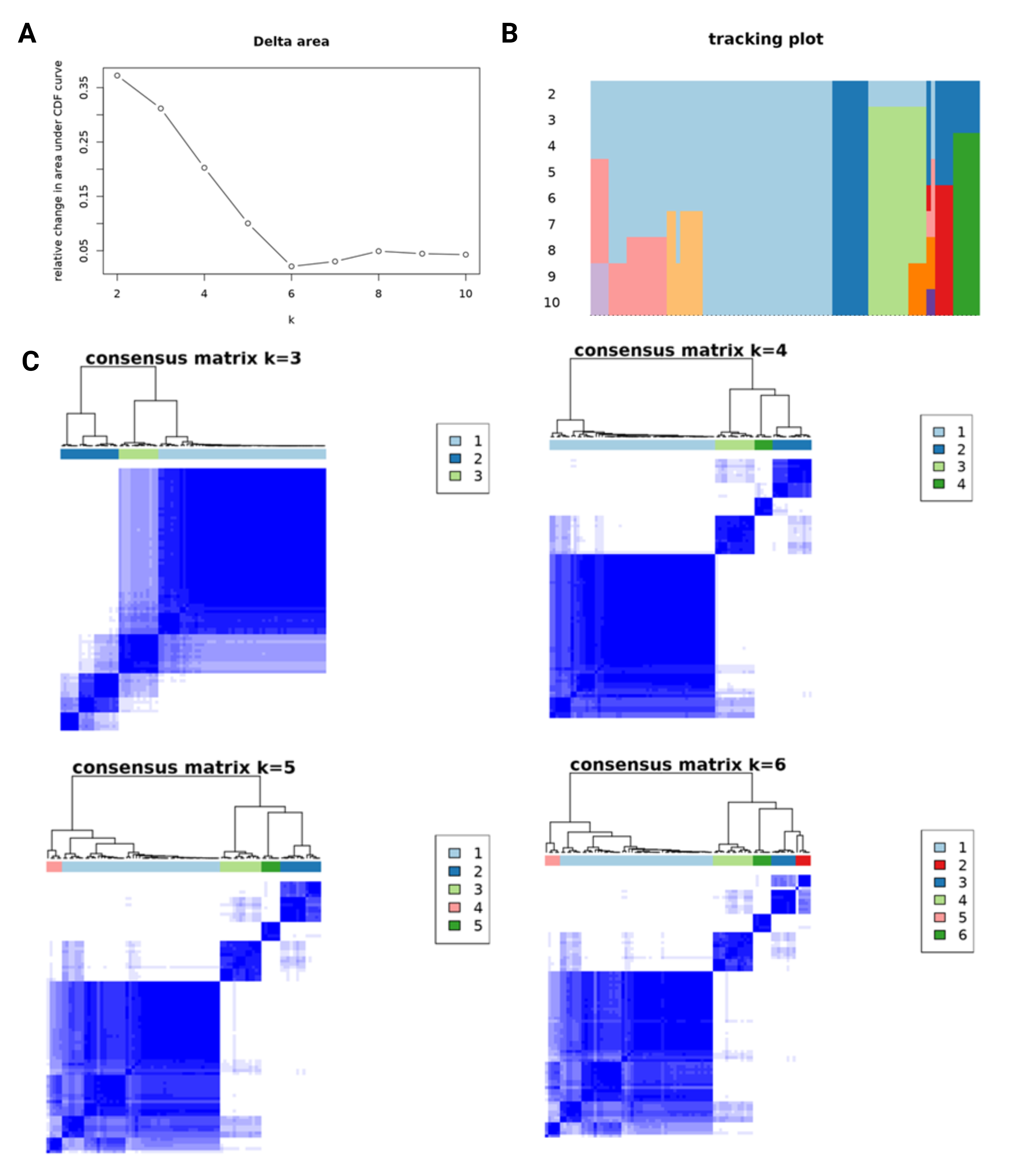

Supplement: cvag089_Supplementary_Data [file cvag089_supplementary_data.zip › V2 Sup Figure 2. Unsup Clustering.jpeg]

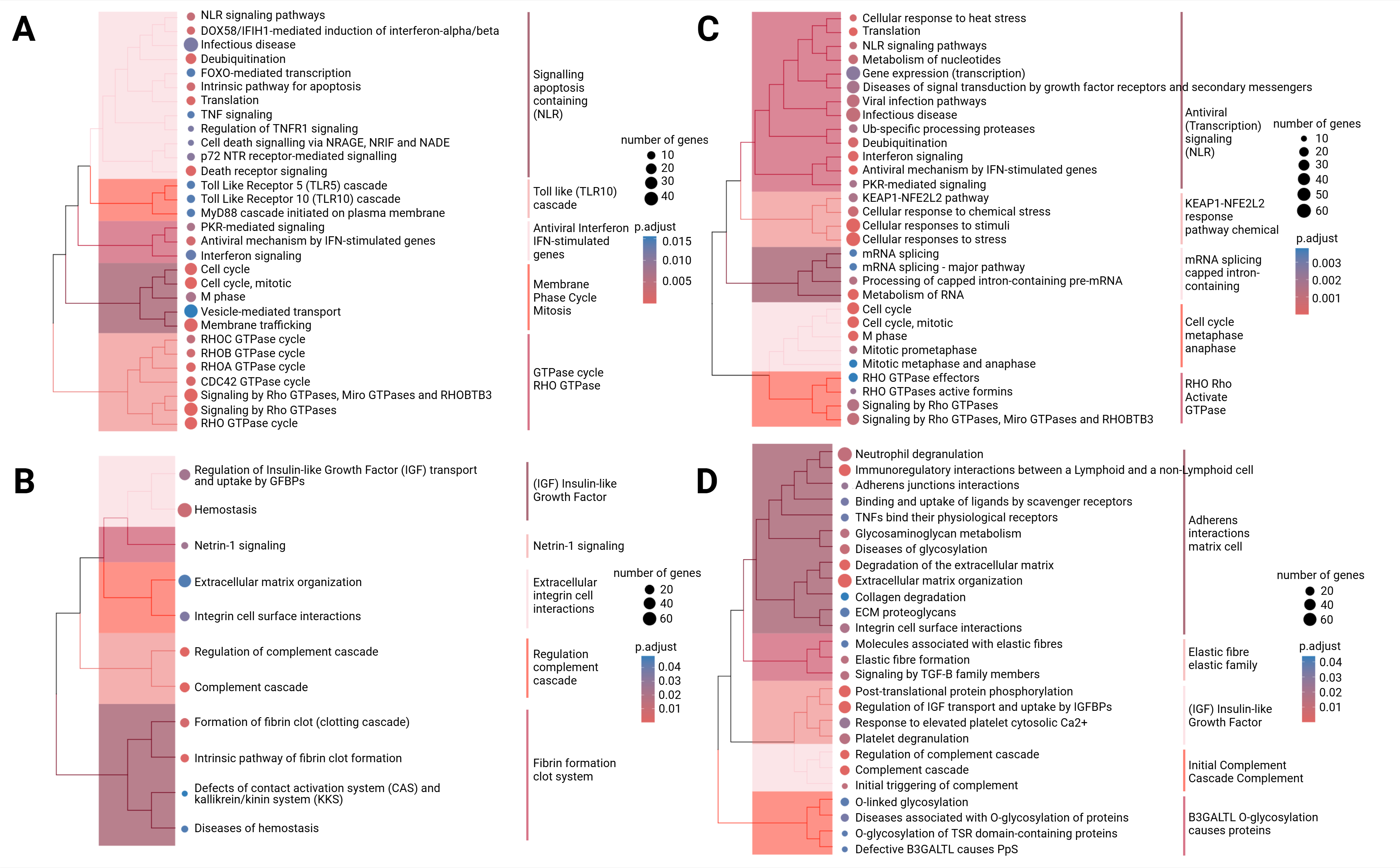

Supplement: cvag089_Supplementary_Data [file cvag089_supplementary_data.zip › V2 Sup Figure 3. Unsup Clustering.jpeg]

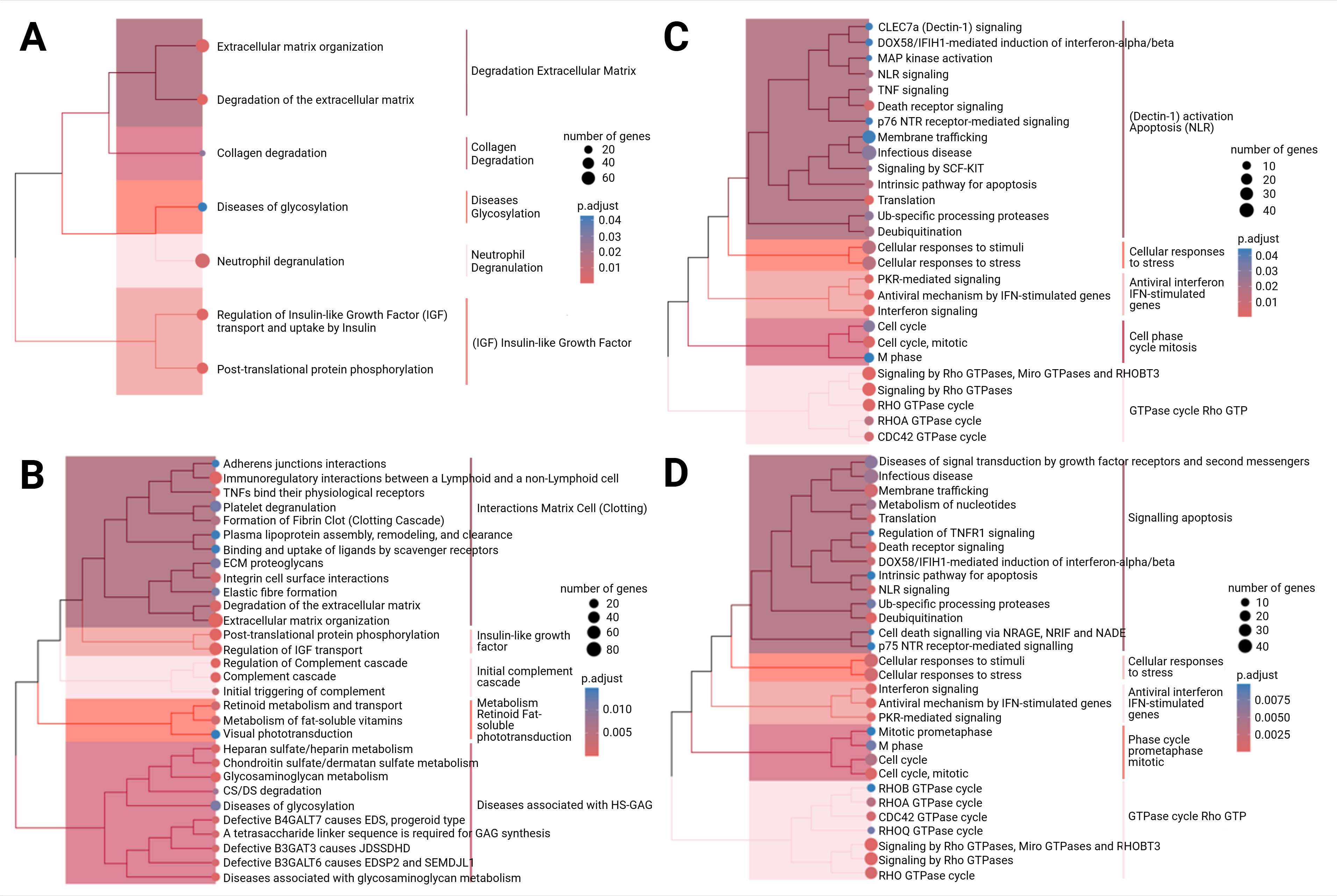

Supplement: cvag089_Supplementary_Data [file cvag089_supplementary_data.zip › V2 Sup Figure 4. Unsup Clustering.jpeg]
